# Spatial and temporal analysis of cumulative environmental effects of offshore wind farms in the North Sea basin

**DOI:** 10.1038/s41598-021-89537-1

**Published:** 2021-05-12

**Authors:** Laura Florentina Gușatu, Stefano Menegon, Daniel Depellegrin, Christian Zuidema, André Faaij, Claudia Yamu

**Affiliations:** 1 Department of Planning, Faculty of Spatial Sciences, University of Groningen, 9747 AD Groningen, The Netherlands; 2 CNR-National Research Council of Italy, ISMAR-Institute of Marine Sciences, Castello 2737/F, 30122 Venice, Italy; 3 Renewable Energy Group, College of Engineering, Mathematics and Physical Sciences, University of Exeter, Cornwall Campus, Penryn, UK; 4 Faculty of Science and Engineering, University of Groningen, 9747 AD Groningen, The Netherlands

**Keywords:** Climate sciences, Ecology, Environmental social sciences, Ocean sciences

## Abstract

The North Sea basin is one of the busiest maritime areas globally with a considerable number of anthropogenic pressures impacting the functioning of the marine ecosystem. Due to growing EU ambitions for the deployment of large offshore wind farm projects (OWF), as part of the 2050 renewable energy roadmap, there is a key need for a holistic understanding of OWF potential impacts on the marine ecosystem. We propose a holistic Cumulative Effect Assessment methodology, applied using a geo-spatial open-source software, to assess impacts of OWF related pressures on selected seabed habitats, fish, seabird and mammal species. We take into account pressures specific to the three OWF development phases, spanning 1999–2050, for the entire North Sea basin. Our results underline 2022 as the peak year of cumulative impacts for the approved OWFs, followed by a considerable increase in potential impacts of the planned 212GWs, by 2050. The spatio-temporal analysis of the OWF environmental impacts presents the shift between highly impacted areas over the studied timeline and distinguishes between concentrated areas of high impacts (S–E of UK) and dispersed areas of high impacts (Germany). Our results can inform decision-makers and the OWF industry in a joint effort to mitigate the environmental impacts of future large OWF developments.

## Introduction

With an increased urgency to reach the 2050 energy targets (United Nations, 2015), the North Sea countries have scaled up their efforts in coordinating and supporting the plans for offshore wind farms (OWF) development^1^. This translates into an extensive deployment of activities related to the construction, operation and lastly decommissioning of offshore wind farms at a marine basin scale, over a long period of time. An extensive deployment of OWF, both geographical and temporal, raises the concern of an increase in cumulative pressures on the already notably impacted marine ecosystem of the North Sea basin^2^, as underlined in numerous national level studies^3–5^. Up to this point, there has been uncertainty related to inconclusive guidance, inconsistent definitions of scopes, and potential cumulative impacts of pressures from multiple OWF developments. These uncertainties have caused delays in the authorization process^6^ (e.g. search sites, such as the Round 2 sites in the UK EEZ, were delayed by over three years)^7^. This could reduce the marine renewable energy developments investor’s confidence, further impede the ability to reach the 2020, 2030 or 2050 EU energy targets^8^, and therefore prolong the societal dependence on fossil fuels and delay the reduction of ${CO}_{2}$ emissions in the environment.

As part of the authorisation process, the individual OWF developments are subject to a systemised assessment of pressures exerted on the marine ecosystem, through the Environmental Impact Assessments (EIA)^9^ or Cumulative Impact Assessments (CIAs)^7^. However, despite the rapid increase in the last years of the OWF deployments in the North Sea basin^10^, as well as the commitment of EU states to maintain these efforts beyond 2030, up to this point no basin spatial scale and long-term assessments of simultaneous OWF developments has been realised.

Enlarging the scope of environmental assessment to the North Sea basin scale, comparing outputs from different OWF Environmental Impact Assessments becomes challenging primarily due to the different EIA methodological approaches used in the North Sea countries^8^, either from a spatial or temporal perspective. The first challenge is related to the allocation of spatial impacts to a specific area, within the current EIAs. Jurisdictional boundaries set obvious spatial scales within which regulatory agencies can enact management measures. Across these measures, however, there is a mismatch between the spatial scales at which ecosystems function, as well as between the impacts of OWFs. This mismatch between spatial scales causes problems when a species is managed locally or regionally, while in reality, the impacts can be the results of activities occurring at an international and thus cross-jurisdictional level^11^. Moreover, the representation of the marine ecosystem at a basin level becomes increasingly relevant particularly due to the transboundary nature of anthropogenic activities, such as OWF. The final aspect that creates a spatial challenge is that the abundance of species is allocated to areas based merely on survey data collected at distinct locations and moments in time, which are used as indicators. This practice also overlooks the high mobility of the marine species^12,13^. A possible response to these mismatches is the identification of suitable reference species, relevant to the North Sea ecosystem, and the assessment of ecological effects on the species population level^14^, through robust methodologies in order to provide a consistent and accepted baseline^15^. A holistic and dynamic approach would imply a representation of the species population distribution at the entire basin level, collating both survey data and predictive models^16^, using basin-relevant data repositories.

In 2008, Halpern et al.^11^ developed the first methodology that relied on assessing the cumulative impacts of human activities on the environment at a holistic (international) spatial scale and was represented spatially through maps^11^. On the regional level, the application of this methodology was presented in a study by Andersen et al.^17^, with a detailed analysis of the combined Norwegian, Danish, Swedish, and German Exclusive Economic Zone (EEZ). Over the last few years, expanding on the initial work of Halpern et al., an increased focus has been given to developing a range of geo-spatial tools as a response to the challenges of quantifying the cumulative impacts of anthropogenic pressures on the marine ecosystem (e.g., Tools4MSP, SYMPHONY, InVEST Habitat Risk). While these tools help create a more holistic perspective and are not limited by addressing the environmental impacts of single wind farms or within distinct jurisdictional boundaries, they do not include the temporal aspects of OWF developments.

The second methodological challenge for an integrated assessment of environmental effects from OWFs across the North Sea basin addresses the different time scales of the OWF-related impacts^14^, as well as the need for a long-term monitoring of effects across the OWF development timeline. The different phases in the life cycle of OWF developments, conducted simultaneously in the North Sea basin, each exert different pressures and impacts on the environment over different periods of time. The phases are typically divided as the following: construction, operation and decommissioning phases. Ideally, a cumulative assessment would rely on a database of information on the location, spatial footprint, and phase of OWF development areas. Thus far, there is no comprehensive North Sea basin wide OWF data repository containing such information that could be used for Cumulative Effects Assessment (CEA) analysis.

The need to develop more integrated and holistic approaches to evaluate the cumulative impacts of OWF over a larger period of time is further raised due to the national development plans that reach beyond 2030 and potentially up to 2050 in the North Sea basin. As a result of this, there is an increased need to monitor the long-term potential effects, not only for the more impactful phases (construction, decommissioning) but also for the long-term, less impactful phases such as the operation phase. While there is currently a good knowledge on the short-term effects of OWF on the marine ecosystem, the long-term effects are insufficiently considered or included in the minimum set of legal requirements^18^. Also, for a basin scale assessment, there needs to be a common set of OWF related pressures considered, as currently the individual EIAs of OWF in the North Sea countries are using different sets of anthropogenic pressures^14^.

In response to both the spatial and temporal challenges, this research aims to analyse the cumulative environmental effects of OWF development in the North Sea basin in the period 1999–⁠2050. More specifically, we aim to: (1) develop a spatial data repository for OWF development in the period 1999–⁠2050 comprising technical details and the status of individual OWFs (construction, operation, decommissioning) per year; (2) compile a data base for the spatially explicit distribution of selected environmental components, including the distribution of fish biomass resulted from a predictive model; (3) collect data from experts’ judgements of specific sensitivities of the selected marine species to each pressure, relative weight and maximum area of influence from the source for the selected pressures; and (4) perform a spatio-temporal assessment for cumulative environmental effects (CEA) of OWF development prospects in three key stages, namely 2020(status-quo, mainly construction), 2030 (EU energy targets benchmark year, mainly operation), 2046 (mainly decommissioning), using a GIS-based open source modelling software. Results from the analysis are expected to support decision-makers and planners in the development of long-term marine conservation and protection strategies, which take into account spatial and temporal patterns of OWF environmental effects in the North Sea basin.

## Results

### Offshore wind farm prospects in the North Sea

**Figure 1:**
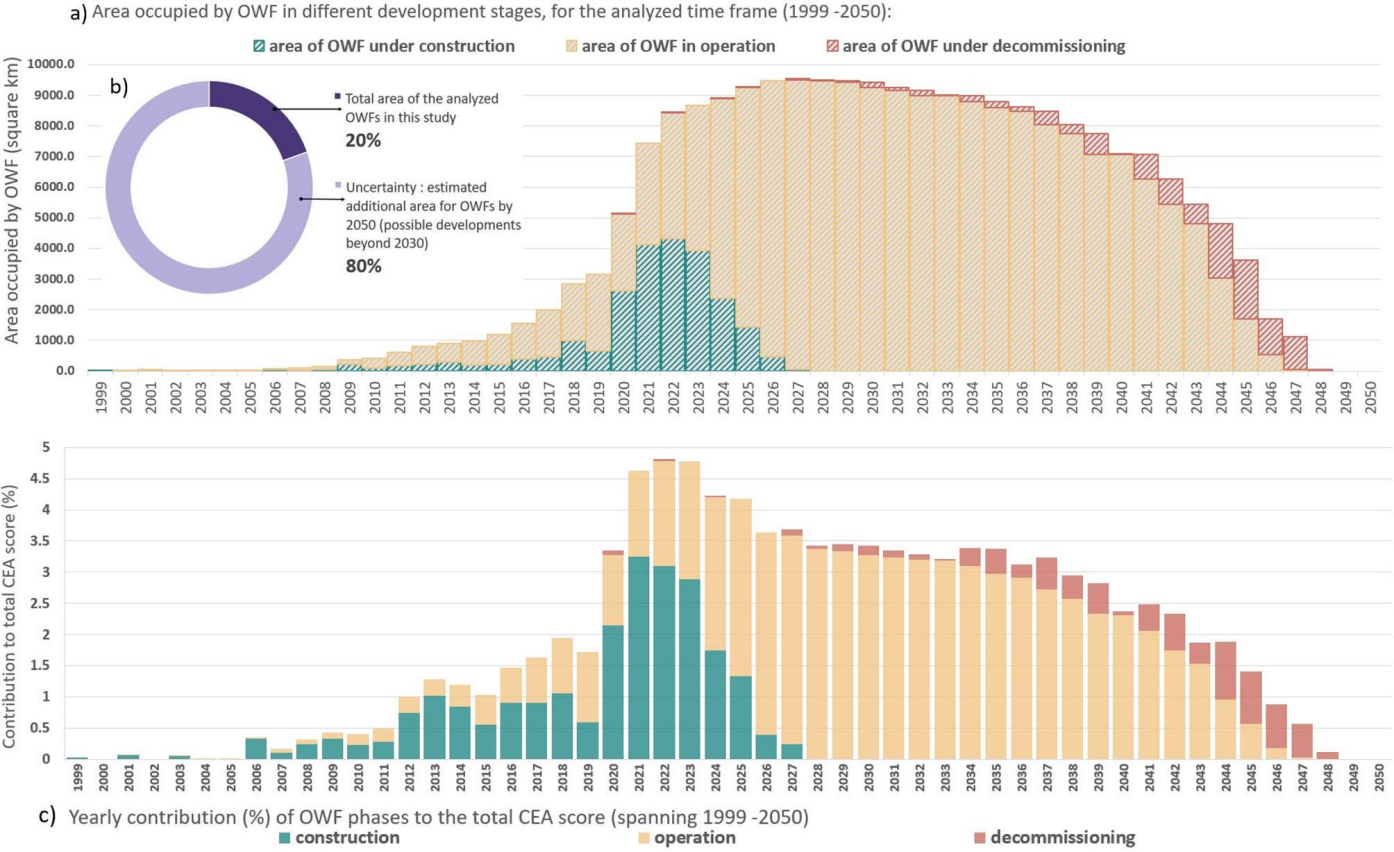
(**a**) Area occupied by OWFs in different development stages, for the analysed time frame (1999–2050); (**b**) estimated additional area for OWFs by 2050 (search areas, development areas, scoping areas for deployments beyond 2030)—uncertainty; (**c**) yearly contribution (%) of OWF phases to the total CEA score (spanning 1990–2050).

The deployment of the OWF in the North Sea basin is an ongoing process that started in 1991 with the installation of the Danish OWF Vinderby, with a total capacity of 4.95 MW, generated by 11 turbines, of 450 kW each ^19^. Since then, the installation rate of OWF has had a positive year-on-year trend^10^, reaching a cumulated installed capacity of approx. 20 GWs in 2020 (79% of Europe’s offshore installed capacity)^20^. Furthermore, several scenarios are indicating an exponential increase in the installed capacity, between 180 GW^21^ and 212 GW^22^ by 2050. An overview of the area required for the authorised OWFs (consent-authorised, authorised), those in the construction phases (pre-construction, under construction) or those in operation (fully commissioned) OWFs in the North Sea basin (Fig. 1) illustrates a continuous development in almost all of the studied countries, until 2048 (based on available data^23^). The geo-spatial repository (Appendix A, Tables 4, 5, 6, 7, 8) we compiled provides information related to the OWFs for which the status of the start date of construction/operation is known (N-3.5 and N-3.6 OWFs in the German EEZ). Therefore, 2027 was the last year considered for construction activities. The area occupied by the OWF developments in the North Sea basin emphasises the increase from 0.4 ${km}^{2}$ in 1999, with the development of Blyth OWF (UK_1, Table 7, Appendix A) in the UK EEZ, to a total of 9577 ${km}^{2}$ in 2027. The total area occupied by the studied OWF represent approximately 1.8% of the Greater North Sea ecoregion, except for Belgium EEZ, Kattegat, the English Channel, as well as estuaries and fjords.

### Spatio-temporal analysis of CEA

The highest proportion of the impacts up to 2023 is associated with pressures from the construction phase, which decrease and are replaced by pressures from the operation phase. The operational phase accounts for approximately 69% of the total CEA score for the studied area, compared to 23% and 8% for construction and decommissioning, respectively. However, relative to the total number of years needed for the operation-related activities, the construction and decommissioning activities have stronger impact. While the construction start date is fixed and the operation start date is considered in the planning of the project (although delays might occur), the start date for decommissioning would occur at the end of OWF lifetime, which in this study is considered as 20 years.

To analyse the contribution of the three phases in different years (Fig. 1b), we compared 2020 and some of the years in the period 2028–2032. While the CEA scores in the compared years are relatively equal, the construction phase contributes to more than 50% of the CEA score for 2020, while after 2027 mainly operation-related activities are contributing to the CEA score. Further clarification can be obtained by also comparing the total area occupied by OWF-related activities in the compared years (Fig. 1a). The total OWF area for any of the years within 2028–2032 period (mainly operation) is approximately double the OWF area in 2020 (mainly operation and construction); hence, the considerably higher contribution of construction-related pressures to the total CEA score compared to the operation phase. Additionally, considerably more intensive additional construction-related activities are expected from 2027 onwards (Fig. 1b), which indicates that the projected CEA score could well exceed the current highest score for 2022. What is currently presented as a stable situation in the interval 2026–2025, with yearly operation-related activities roughly three times more impactful than in the year 2020, would experience a substantial increase in impacts (Fig. 5).

**Figure 2:**
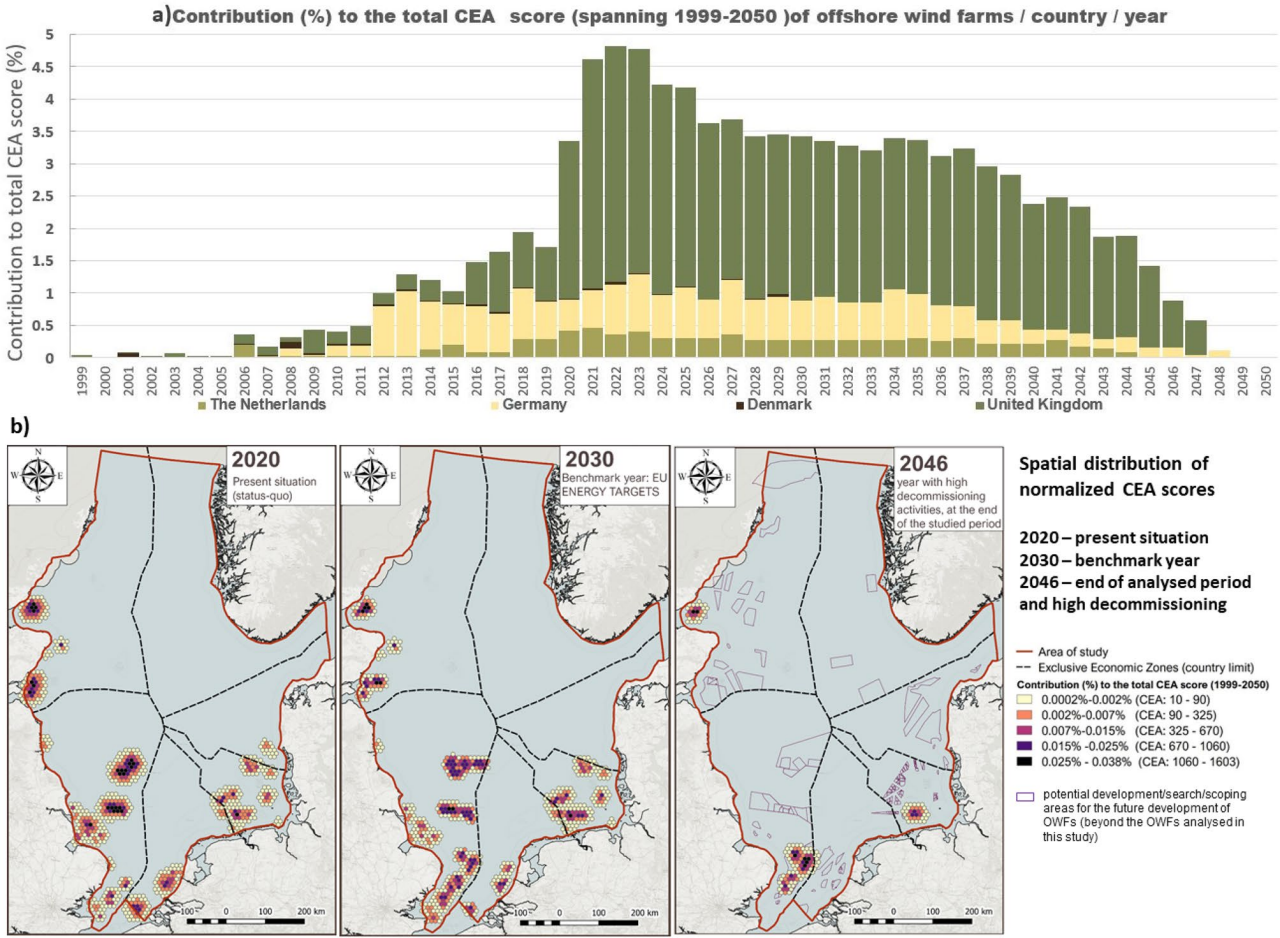
(**a**) CEA scores of offshore wind farms in the analysed countries for 1999–⁠2050; (**b**) geospatial distribution of the CEA scores (2020, 2030, 2046).

The distribution of CEA scores over the 1999–⁠2050 timeframe (Fig. 2a) underlines an increase in the cumulative OWF impacts in the maritime space of Germany, Denmark, the United Kingdom and the Netherlands, up to 2022. The range of yearly impacts variates from the year 2000, the year with the lowest impact of 0.003% CEA score, to the year 2022, the year with the highest impact of 4.81% CEA score. The plot also shows the constant rate of decrease of impacts in all of the analysed countries, towards the end of the studied period. This, however, will significantly change in the likely event that parks, yet to be planned or agreed upon, will be developed by then. When differentiating the contribution to the CEA score per country for the entire timeline, a clear role is played here by the OWF located in the UK maritime area (70.6% total CEA score), followed by Germany with a considerably lower percentage (20.3%) and Netherlands (8.4%). In comparison, Denmark brings a minor contribution to the cumulative impacts, with 0.7% CEA score across all development stages.

Figure 2 illustrates the temporal variation of CEA scores across the analysed area of study. The status quo (2020) of CEA impacts underlines an uneven distribution of impacts between the EEZ of North Sea countries, with the highest scores in the British EEZ corresponding to the location of OWFs Sofia (UK_41), Dogger Bank A (UK_39) and Hornsea 1 and 2 (UK_33, UK_34). The map illustrating the CEA scores in 2030 displays multiple hot spots of intensified impacts, mainly from localised pressures from the operation phase of OWF. Towards the end of the analysed period, the highest contribution to the cumulative OWF impacts on the marine environment are produced by high decommissioning related activities, one exemplifying year being 2046. The impacts presented at the end of the analysed period are however underestimated (Fig. 5, “Discussion” section), since more OWF areas are planned to be developed, as illustrated on the 2046 map.

**Figure 3:**
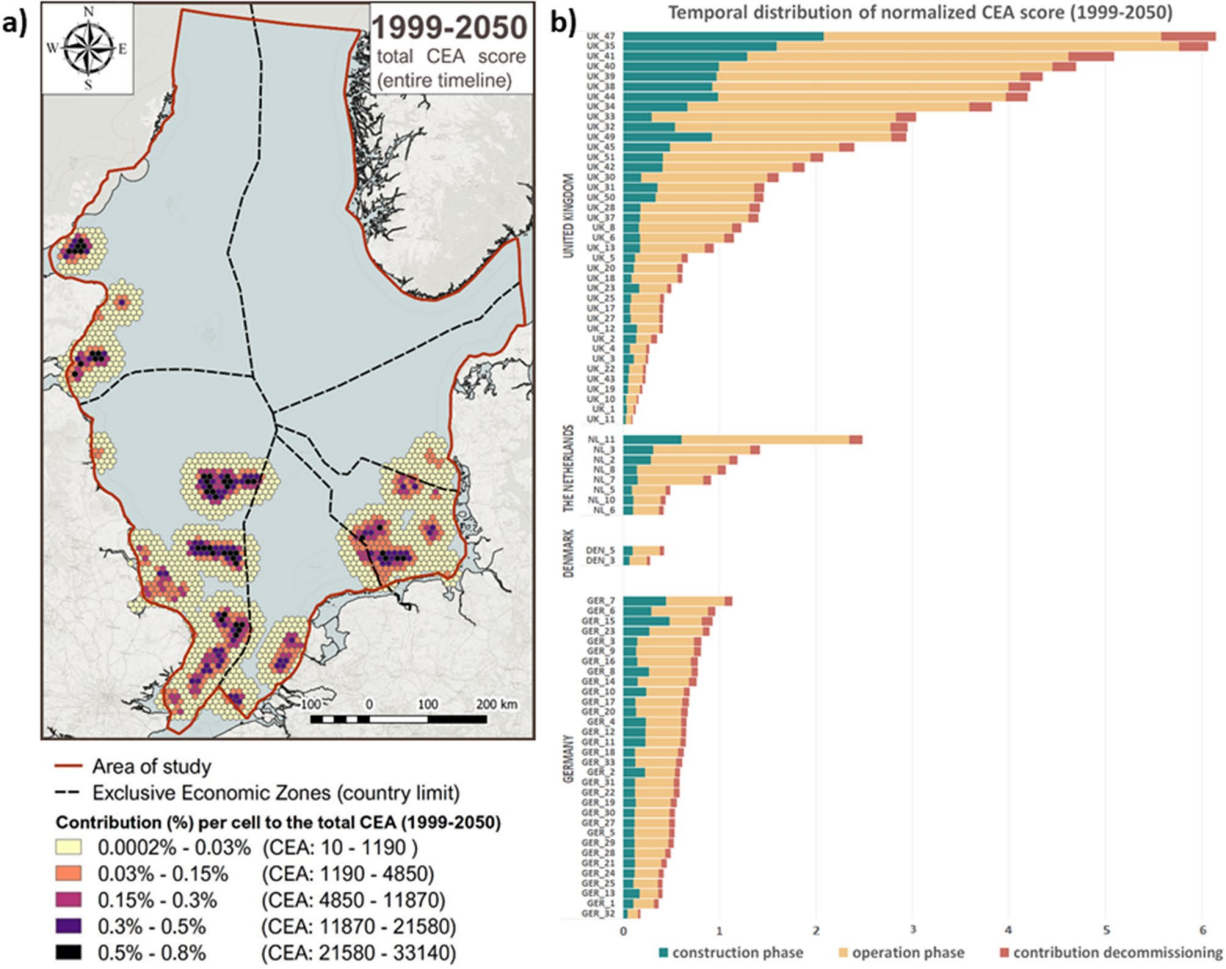
(**a**) Spatial distribution of the total CEA scores for the entire timeline (1999–⁠2050); (**b**) temporal distribution of CEA score (1999–2050) per phase.

In the analysed period, the minimum individual OWF impact is 0.09% CEA score and would be generated by the Gunfleet Sands 3 OWF (UK_11, Fig. 3b), located in the southern part of the British EEZ, which has a production capacity of 4 MW that is generated by 2 turbines. In contrast to this, the highest level of impact of 6.15% CEA score would be generated by the Norfolk Vanguard OWF (UK_47, Fig. 3b), which is located on the S–E side of the British EEZ and has a production capacity of 1800 MW that is generated by approx. 180 turbines.

### Spatio-temporal representation of high cumulative effects

An overview of the total CEA scores, for the entire timeline (1999–⁠2050), illustrated in Fig. 3a, underlines the main concentrations of OWF related impacts, with the four most impactful clusters localised in the German EEZ and British EEZ. A distinction can be made between cluster areas of spatially concentrated high CEA scores (0.3–0.8%; north-east and central British EEZ) and dispersed wide areas of high-medium CEA scores (0.03–0.8%; south of British EEZ and German EEZ) (Fig. 3a). While there is not a significant variation between the added CEA scores/cluster of each of the two categories implying their total impact is fairly similar (concentrated: 18.35% and 13%; dispersed: 16.6% and 15%), special attention needs to be given to the area of impact. The two clusters of concentrated high impacts (adding 4 and 3 OWFs) affects approximately 8583 ${km}^{2}$, of which 50% (4248 ${\mathrm{km}}^{2})$ with high CEA per cell (0.3–0.8%). On the other side, the two clusters of dispersed high-medium impacts (7 and 22 OWFs) affect approximately 13,438 ${\mathrm{km}}^{2}$, with the same number of high CEA scores per cell (0.3–0.8%) but almost three times more cells of medium and low impact per cell (0.03–0.3%).

### Pressures and environmental components

Figure 7 illustrates the impact chain of each phase-specific OWF pressure for the respective environmental components, where the strengths of the links are relative to the level of sensitivity of the species to the respective pressures. Based on a literature review and validated through expert questionnaires, our study has identified the pressures to which the selected environmental components are most sensitive: underwater noise, habitat loss, and risk of contact with fuel or chemicals. The lowest species sensitivity is related to heat effect due to cabling, change in hydrodynamic regime, change in physiochemical water quality, and collisions (with vessels or turbines). In terms of spatial magnitude, the construction phase tends to have a large area of impact through pressures such as underwater noise and marine litter, while the operation phase is characterised mostly by localised pressures (Fig. 8).

**Figure 4:**
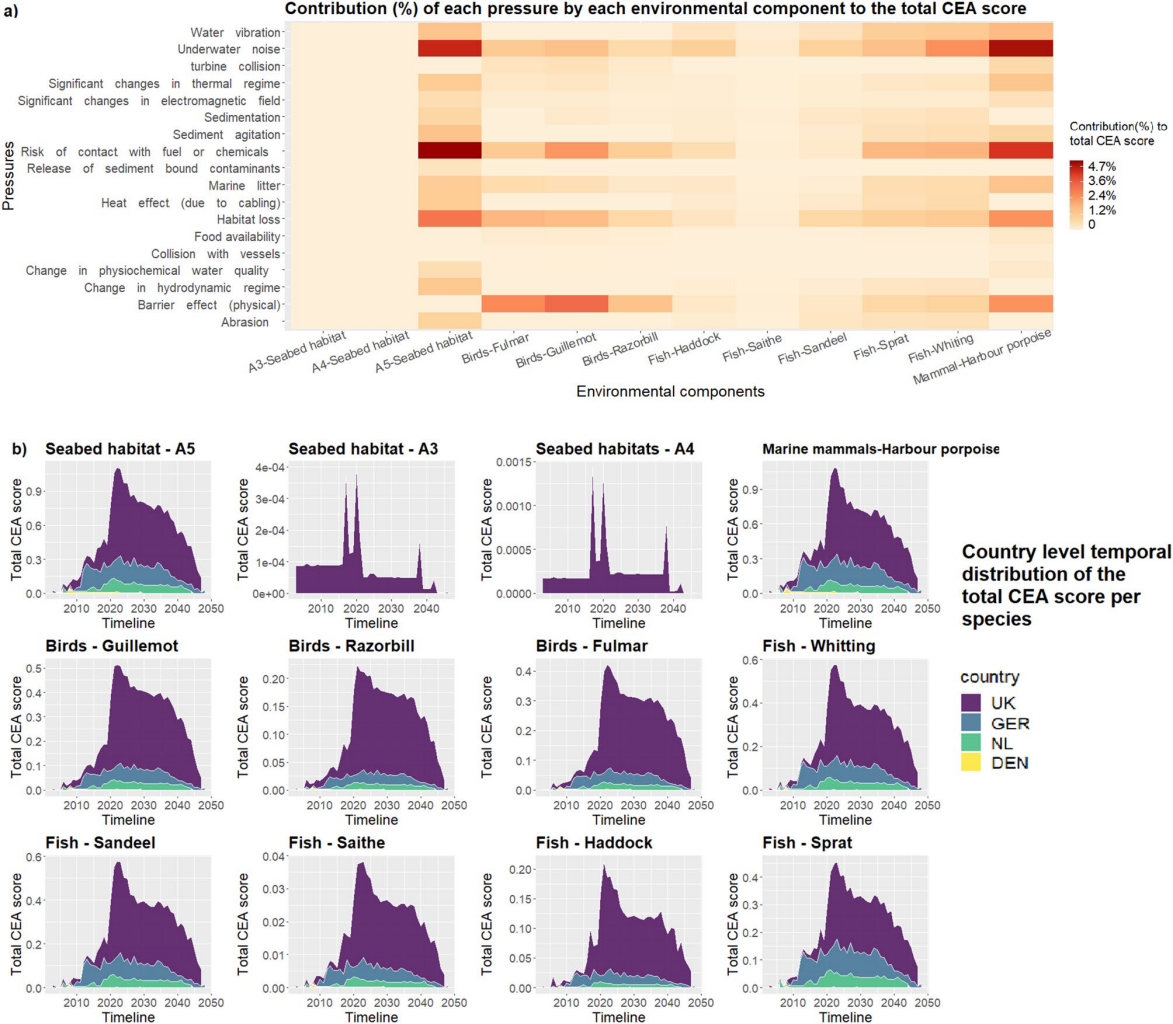
(**a**) Contribution (%) of each pressure by each environmental component to the total CEA score; (**b**) country level temporal distribution of the contribution of pressures to the total CEA score (1999–2050), by country, for the analysed environmental components.

Figure 4a reveals the contribution of the 18 pressures exerted by OWF to the CEA for each of the 12 environmental components analysed. Pressures with high impacts on the analysed species and habitats are habitat loss, barrier effect, underwater noise and risk of contact with fuel and chemicals. Underwater noise has the highest impact on the harbour porpoise (4.7%), followed by A5—sublittoral sediment (4.5%), and whiting (2.5%). The barrier effect mostly impacts guillemot (3.3%) and fulmar (2.6%), but also the harbour porpoise (2.4%). The risk of contact with fuel and contaminants impacts mainly the A5—sublittoral sediment habitat (4.7%), and the harbour porpoise (2.5%).

Figure 4b illustrates the higher contribution of the UK OWFs to the CEA scores for all analysed environmental components. The temporal distribution of pressures indicates a similar pattern of impacts on all the analysed species and habitats located in the UK EEZ, underlining 2020–2025 as the peak period of impact. More variation in impacts can be seen in the case of fish species in the German and Dutch marine spaces.

The sensitivity analysis performed on the effect function and pressure propagations (Appendix B) showed that during the OWF construction phase highest sources of uncertainty for pressure propagation are marine litter (8.5%) and synthetic compounds (8.2%). Highest uncertainty on effect functions refer to noise on sub-littoral sediments and the marine mammal Harbour porpoise (7.7% and 7.6% respectively). In the operational phase, most uncertainty refer to synthetic compounds (15.3%), sealing (14%), release sediment bound contaminants (13.6%). In the decommissioning the most uncertainties refer to marine litter (18%), noise (8.7%) and synthetic compounds (7.8%).

## Discussion

This study analysed the CEA of OWF developments on the North Sea for the time period spanning 1999–2050. For the geospatial analysis of the cumulative effects, we used the open source software, Tools4MSP, over the entire timeline of the decommissioned, authorised, under construction or operational OWFs in the North Sea basin. We first presented the peak years and the regions with the highest contribution to the total CEA score. For a better understanding of the spatio-temporal changes in the intensity and location of impacted habitats, we mapped the distribution of the CEA scores in three key years (status quo, EU energy targets benchmark year, year with high decommissioning activities). Following this, we summarised the individual CEA score for each species over the lifespan of the analysed OWFs, by country.

**Figure 5:**
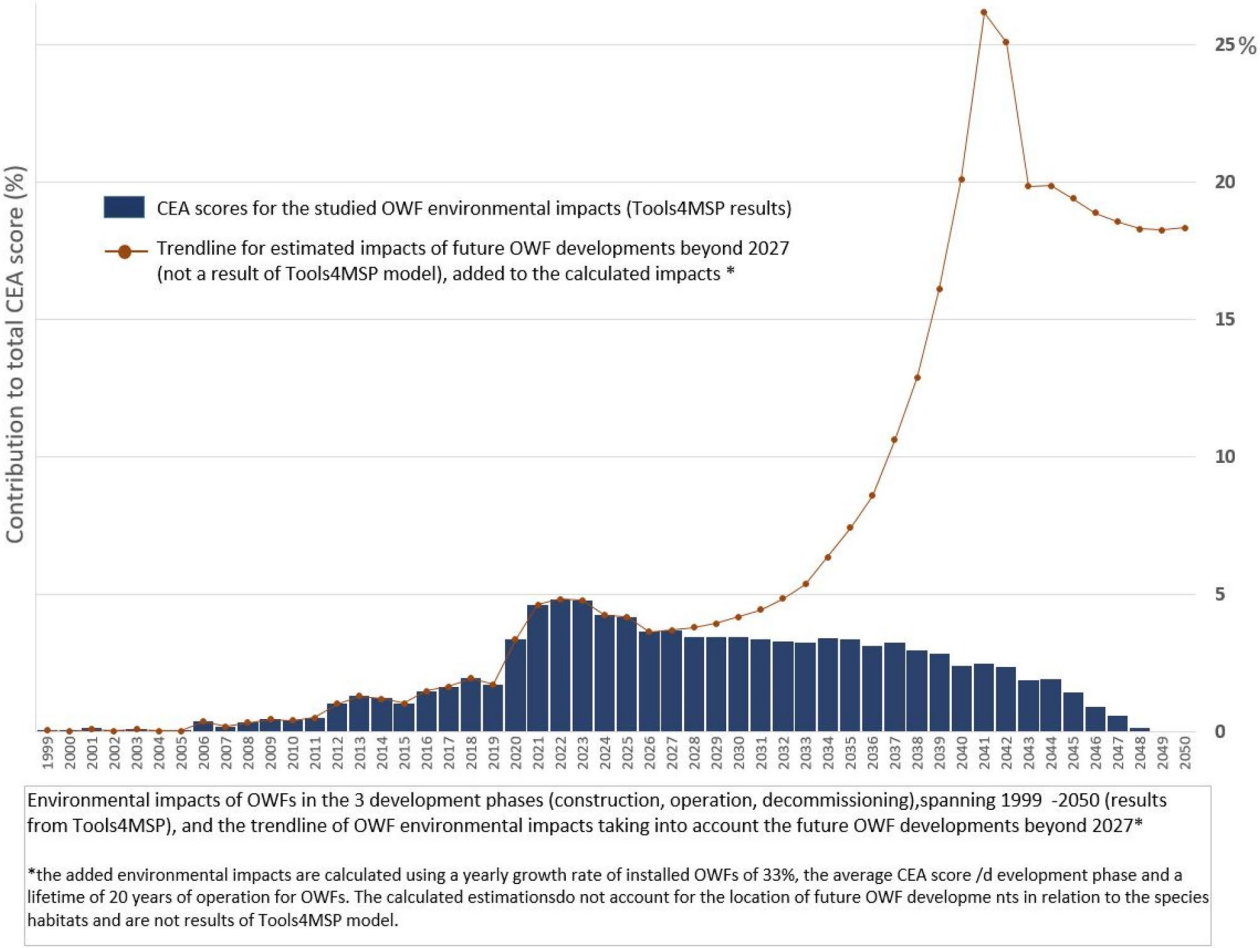
Yearly contribution (%) of OWF phases (construction, operation, decommissioning) to the total CEA score, spanning 1999–2050 (results from Tools4MSP), and the estimated impacts of future developments (beyond 2027, last year of analysis).

Furthermore, we made a distinction between groups of OWFs with high CEA scores, concentrated in a relatively small area (southern part of the British EEZ), as opposed to groups adding a large number of OWFs, with a high total CEA score, dispersed over a wider area (German EEZ). The temporal distribution of CEA scores reveals high CEA scores for OWF construction in 2021, for OWF decommissioning between 2044 and 2046, and for the operational OWFs between 2026 and 2037. This is due to the fact that we only included OWFs that were either under construction, operational, or had been formally agreed upon at the time of this study. Therefore, all OWFs that might be developed beyond 2027 that have not yet received formal agreement were not included, neither were their impacts. However, approximately 40,000 ${\mathrm{km}}^{2}$ are still under development or scoping for locating future OWFs beyond 2027 (Fig. 5), which represent at least four times the area analysed in this study. This would add a consistent number of impacts to the identified cumulative scores, considering the scaling of OWF developments from the currently analysed 41.58 GWs to the planned 61.8–66.8 GWs by 2030, and later to 212 GWs by 2050. Figure 5 illustrates a potential scenario of added cumulative OWF impacts beyond 2027, based on the average yearly growth rate of the installed GWs between 1999 and 2022 (33%), the EU target of 212 GWs by 2050, the average operation time for OWFs (20 years) and the average CEA score/GW for each of the OWF phases, using the results of this study. Taking into account solely the impacts of the construction phase, continuing the deployment of OWFs up to 2050 at a similar pace would significantly change the timeline of impacts presented in Fig. 5. This illustration, however, represents only one scenario of a large range of possibilities since the CEA score is highly dependent on the location of the future sites in relation to the distribution of the marine species.

The environmental effects analysed in this study focused on the negative impacts of OWF activities, from the lens of recent reports and studies, validated through expert questionnaires. Consistent with previous studies, the cross-phase analysis highlights that the most impactful pressures occur during the construction phase, in particular the underwater noise. As underlined by interviewed experts, even if in the operation phase the underwater noise has a lower impact compared to the construction phase, it is a continuous disturbance taking place over a large period of time. This is particularly relevant since the operation phase accounts for 69% of the impacts calculated in this study, indicating a temporal accumulation of disturbances in continuous, potentially irregular manner, spanning 1999–2050. As emphasised by the expert-based questionnaires, this could affect the analysed species differently, such as the reproduction and communication between groups of fish, the hearing of predators, or the settlement in the area, but also influencing the ecological connectivity and interdependencies between important environmental elements^8^. For the migratory birds, the alignment of the OWF development related activities to the seasonality of the seabirds is essential in maintaining the connectivity between colonies, areas of foraging and breeding areas. One example in this case is the Special Protection Area (SPA) Friese Front, located in the central part of the Dutch EEZ, designated for the sleeping and resting place for Guillemots^24^. Other relevant examples are the Special Area of Conservation (SAC) Voordelta^25^, the SPA Vlakte van de Raan^26^ and the area of special ecological value Zeeuwse Banks, foraging and resting areas for over 30 bird species, marine mammals such as harbour porpoise and gray, located in the close proximity of Borselle 1,2,3 and 4 OWFs, in the S–W of the Dutch EEZ. In maintaining the favourable status of the protected species, the objective of these protected areas, the management of the approved and proposed OWFs site would benefit from an assessment of the timeline and intensity of OWF related pressures. Therefore, even if the construction phase has the highest contribution to the total CEA (1999–2050), particular attention should be given to the concentration of more localised but continuous pressures specific to the operation phase.

The intensity of OWF impacts on the species’ habitats (Fig. 4a) reveals the spatial relationship between the distribution of OWF areas and the distribution of the selected species. Therefore, the very low CEA scores for the circalittoral rock (A4), Infralittoral rock (A3) or low CEA scores for fish species such as sandeel should not only be explained through the sensitivity of those environmental habitats to OWF pressures, but also through the spatial interaction between the analysed habitats with the analysed OWF areas (presence/absence or distance from the source of pressure). Moreover, while CEA scores by environmental components cannot be compared across species groups, the overview of the temporal distribution of impacts per country clearly differentiated the high impacts resulted from OWFs located in the British EEZ, for all selected species.

The outcomes of the study are showing important potential to support decision making processes for the planning of future OWF developments, in particular when considering the substantial additional OWF related impacts expected from OWFs deployed up to 2050. Policy aiming to protect, maintain or improve the ecological functions or the environmental components of the marine ecosystem can benefit from understanding the different spatial and temporal patterns of accumulated effects of OWF related pressures^6^. One example is managing the development of OWFs in relation to the current status and the conservation objectives for the protected features (habitats, marine species) in the Special Protection Areas (SPAs) or Special Areas of Conservation (SACs). Taking into account the current unfavorable status of harbor porpoise in the Voordelta SAC and the conservative objective to restore the population, measures to minimize the most impactful OWF related pressures, namely underwater noise and risk of contact with fuel and contaminants (Fig. 4a), should be prioritized in the development stages of near OWFs (Borselle 1,2,3,4 OWFs). The results of the study can therefore be further used in the context of relevant environmental policy objectives, as a CEA step of risk evaluation for the management procedures regarding potential failure to meet such conservation targets for certain selected species or habitats^27^.

Our results point to the need to consider the timeline and transboundary nature of OWF’s cumulative environmental impacts in the formulation of alternative options for the location of future OWF sites in the North Sea basin. Thus, we presented two different OWF planning approaches with similar contributions to the total CEA score, namely between a large, dispersed cluster in the German EEZ (22 OWFs) and a small, concentrated cluster in the south of the British EEZ (three OWFs). This highlights the necessity to consider the management of activities and pressures taking place simultaneous within a certain geographical area, also in alignment with the seasonality of selected environmental components (e.g., migration patterns for breeding and foraging). Therefore, the information provided on the key years of high impacts, in addition to the spatial shift in highly impacted areas, could potentially be used by decision-makers and OWF developers in planning the OWF location and timeline of phases. In return, this could minimise the cumulative environmental effects on the marine species.

Furthermore, the results of a large geographical scale and long term assessment model can represent input data for the marine conservation strategies, monitoring of long-term plans for the sustainable development of marine space^2^, Marine Spatial Plans, and multilateral consultations among relevant parties for transboundary projects. The spatio-temporal analysis performed in this study can be used to overcome misleading results of cumulative impact assessments realised at the individual project scales, caused by disregarding the transboundary nature of the marine environment, and therefore of impacts on the marine ecosystem. This enables a knowledge-based, coherent assessment of impact significance on an accurate distribution of the selected species, taking into account transboundary effects. Although there is only a small number of transboundary projects in the North Sea, the interest in those projects has increased over the last decade, and it mainly addresses issues related to the conservation of the marine environment but also renewable energy projects^28^. One tool that can be useful in addressing this gap is the Strategic Environmental Assessment (SEA, https://ec.europa.eu/environment/eia/sea-legalcontext.htm), which has already been applied at the national level to guide the responsible implementation of renewable energy programs^29^. The methodology and workflow based on the Tools4MSP Modelling Framework has the flexibility to be applied at any spatial scale. Applications performed so far include macro-regional level^30^, national level^31^ and applications on single OWE project scale^32^. Depending on the research objective and data quality, spatial scales and resolution can be fully customized by the user. In addition, the workflow enables to perform CEA on other Blue Economy activities (e.g. aquaculture, commercial fishery, oil and gas, shipping) simultaneously. The integration of a temporal dimension into the Tools4MSP Modelling Framework is novel and showed promising applications to investigate single use planning cycles and in future more comprehensive MSP cycles.

The CEA analysis is prone to multiple uncertainties, related to model assumptions or data quality^27^. Here we present the limitations of this study. Firstly, the spatial accuracy in representing the distribution of the selected species could be improved. Additional spatio-temporal features could not only account for the location of breeding, non-breeding areas, and colonies of seabirds, but also the potential impacts of climate change factors in the change of species’ habitats^33^. Despite this limitation, studies addressing the species distribution for the North Sea basin^34,35^ can attest for the robustness of the proposed predictive model. Moreover, as suggested by the interviewed experts, the species database of this study can be further extended for a better representation of the marine ecosystem and the different food chains. Some suggested additions for fish species are the gobies, cod, and herring, while further added seabird species can be the diving duck, common scouter, kittiwake and gannet.

Secondly, other uncertainties can be linked to the quantification of the input parameters, such as pressure importance weight scores, OWF-related pressure propagation distances or the sensitivity scores of the selected environmental components to the identified pressures. Consequently, the expert-based questionnaire includes a confidence level. The applied sensitivity analysis performed separately for each OWF phase (Appendix B) allowed to quantify the sources of uncertainty in terms of assessing pressure propagation and effects on environmental components. This allows to sort and prioritize knowledge gaps in the input data definition, pressure propagation modeling (e.g. underwater noise and marine litter) and address improvements in the expert elicitation process in terms of sensitivity scoring mechanisms.

The numeric scores that resulted from the expert interviews and literature review were aggregated based on the precautionary principle, taking into account the confidence level. Improving the reliability of the input sources would require further increased details on the impact of the specific analysed pressures on the analysed environmental components. The sensitivity scores and the pressure weights used in this study are based on the literature review and expert interviews. Quantification of impacts is, therefore, based on the current knowledge of the technical characteristics of OWF turbines, as well as the current display and densities in the North Sea. In new development areas, different conditions might apply, such as the use of bigger turbines (6–10 MWs), which would lead to different densities and OWF layouts. Also, different parameters influencing the duration of the three development phases could be further considered. For one, the technological advancements of OWFs with a monopile foundation have led to a decrease of the installation time from 2016 to 2017 as compared to the period 2000–2003^36^. Furthermore, the type and duration of the OWF decommissioning is not yet clear. The parameters influencing the decommissioning time are: the number of turbines, the foundation type, the distance to the port, the vessels used for the removal operation, the options for decommissioning (complete removal, except foundations; partial removal due to multiple environmental externalities) and the weather conditions^37^.

While a consistent body of research has focused on species’ sensitivities to high impact, short-term pressures (i.e., underwater noise), there remains a lack of understanding of the effects of exposure to long-term pressures. Additionally, when designating scores to pressures and species sensitivities, certain principles need to be taken into account^11^: environmental effects exerted by human activities can be synergetic and/or antagonistic; not all stressors are equal or have impacts that increase linearly; and the assessment must account for the different geographical scales of activities and impacts. Therefore, this study can not only be further complemented by including pressures from other offshore activities, but also through a clearer differentiation between localised pressures and pressures with larger geographic impacts.

Lastly, within this study we identified only the cumulative negative environmental effects related to the OWF pressures, in different development phases. Therefore, a truly holistic picture of the overall impacts of human activities on the marine environment can be reached by adding the already large environmental impacts of other sea uses, such as oil and gas related activities, commercial fishery, shipping and military activity. One of the most impactful activities on the biomass and biodiversity of the North Sea benthic ecosystem and seabed habitat is bottom fishing^38^. The surface and subsurface abrasion caused by the fishing activities footprint affect large areas localised along the Norwegian coast and southern North Sea, leaving only 7% of the shallow and deep zones in the Greater North Sea untrawled^39^ (Appendix C). In comparison to this, the area subject to the analysed OWF sums 9577 ${\mathrm{km}}^{2}$, for the entire analysed period, which represents approx. 1.8% of the studied area. Hence, a coordinated spatio-temporal analysis of multiple pressures related to offshore activities, and their potential cumulative effects over the marine ecosystem, would provide a complete understanding of the relevant changes in the carrying capacity of the marine environment, the population stability, their resilience and recoverability. The spatial aspects, referring to the localization and the space between the perturbations in relation to the receptors, and temporal aspects, referring to the temporal accumulation of pressures in relation to the recoverability of the receptors, are therefore essential elements of the CEA analysis that can provide input to management alternatives of the marine space^8^.

Moreover, future research on the potential synergies, risks, and trade-offs between the traditional sea users and the future OWF developments can benefit decision-makers and the OWF industry in the planning of future OWF locations, as well as in the monitoring and managing their environmental effects. As also emphasised by the experts interviewed, placing the OWF infrastructure in areas highly impacted by trawling activities could also have a potentially positive effect during the operation phase. This is linked to the potential protection status of OWF areas, which could, in the absence of fishing activities, act as refuge and habitat for juvenile flatfish species on a small geographic scale^40^, as well as areas of community recovery for different commercial fish species attracted by OWF structures^41^. The increase in the complexity of the seabed, formed on the new structures, also presents opportunities for food and shelter to benthic communities^42^, and provides additional food sources for higher trophic levels^43^.

The potential area of protection is of minimum 9577 ${\mathrm{km}}^{2}$ (Fig. 1), which will be further enhanced by the additional surface of the scoping and search OWF areas, beyond 2030. However, the OWF areas can rather be seen as a system of smaller reefs related to single turbines, with a footprint from of approximately 50.3 ${\mathrm{m}}^{2}$ for monopile foundations of 8 m diameter, to approximately 400 ${\mathrm{m}}^{2}$ for a jacket foundation of 20 m diameter^41^. This is derived from the substantial distances between turbines, which can vary from 500 to 1000 m^44^. Furthermore, the epifauna assemblages formed on hard substrata provided by the OWF is different compared to natural reefs^41,45^ because they have the potential to also colonise non-indigenous species^41,46^. Nevertheless, the potentially positive contributions of the new OWF structures to habitat modification, ecosystem processes, and functions, also detailed by Causon et al.^41^, could lead to the ecosystem restoration of the currently degraded marine habitats of the North Sea basin^47^; hence, future research on cumulative environmental effects can benefit from including such potentially positive effects.

Further research is needed to improve holistic approaches to evaluating cumulative environmental effects of OWFs, which should, ideally, at least address the most evident gaps. One example is the recoverability time of specific species, which was not explicitly considered in modelling the temporal aspect of CEA. Other example can be related to the effects of including hard substrate (turbine foundations) in different types of seabed habitats which might impact many processes that sustain a functioning ecosystem. This characteristic was, however, part of the sensitivity score, for which it was described as a function of the ability of the environmental component to tolerate and resist change from impacts.

In conclusion, this study specifically adds value to the existing research on the assessment of cumulative OWF environmental impacts, through a holistic methodology that included both more spatial as well as the temporal detail. The proposed spatio-temporal approach represents a versatile instrument to support the Marine Spatial Planning, the Environmental Impact assessment, and the Strategic Environmental Assessment. Our findings underline the urgency to more systematically manage the growing impacts resulting from ample OWF projects through strategic spatial and temporal planning, for the entire North Sea basin.

## Methods

**Figure 6:**
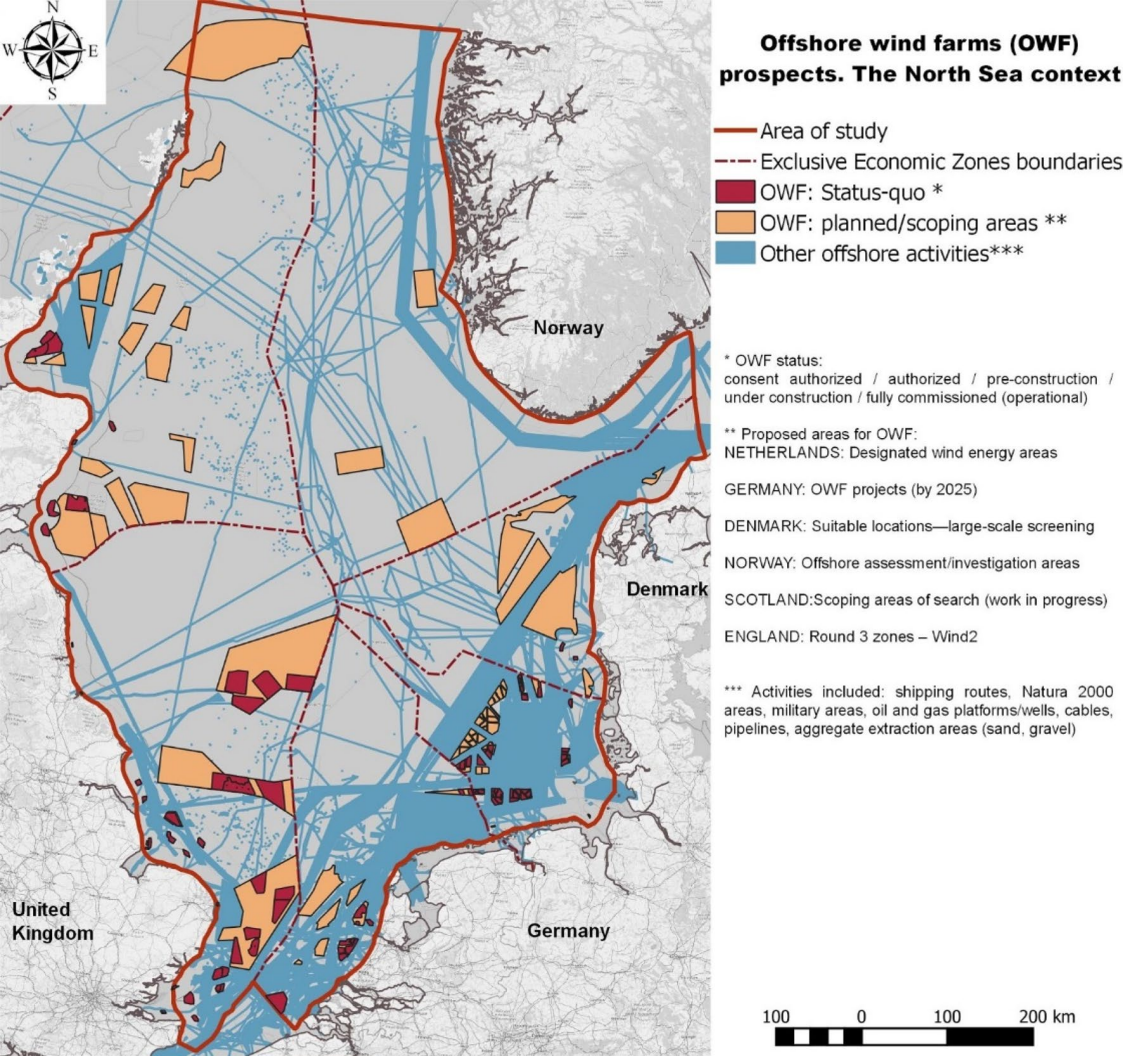
Offshore wind farm prospects (existing/authorised/planned) in the North Sea basin.

The area of study (Fig. 6) was the Greater North Sea ecoregion, which includes the EEZs of six countries (England, Scotland, the Netherlands, Denmark, Norway and Germany). The Kattegat area, the English Channel, and the Belgium EEZ were omitted from the study area. The North Sea Marine Ecosystem is a large semi-closed continental sea situated on the continental shelf of North-western Europe, with a dominant physical division between the comparatively deep northern part (50–200 m, with the Norwegian Trench dropping to 700 m) and the shallower southern part (20–50 m)^48^. The North Sea is one of the most varied coastal regions in the world, which is characterised by, among others, rocky, fjord and mountainous shores as well as sandy beaches with dunes^48^. Apart from the marine seabirds feeding primarily in the coastal areas, under 5 km from the coast (e.g., terns, sea-ducks, grebes), the North Sea basin also hosts pelagic birds feeding further offshore, with some also diving for food (guillemot, razorbill, etc.). The North Sea basin is also a major habitat for four marine mammal species, of which the harbour porpoise and harbour seal are the most common. Moreover, fish ecology has been a widely studied topic, especially for commercial species, due to evidence of a decline in the fish stock, such as sprat, whiting, bib, and mackerel. Fish communities, and in particular the small pelagic fish group (such as European sprat, European pilchard), play also a key ecologic role, constituting the main pray for most piscivorous fishes, cetacean and seabirds^49^, Based on early surveys, the predominant species divided by the three North Sea fish communities are: saithe (43.6% in the shelf edge), haddock (42.4% in the central North Sea, 11.6% in the shelf edge), whiting (21.6% in the eastern North Sea, 13.9% central North Sea), and dab (21.8% in the eastern North Sea)^34^. More recent assessments of North Sea fish community are emphasizing the clear geographical distinction between the fish species living in the southern part of the North Sea, a shallow area with high primary production and pronounced seasonality, and northern part, a deeper area with lower primary production and lower seasonal variation in temperature and salinity. The southern North Sea fish community is represented by fish species such as lesser weever, while the northern North Sea fish community is represented by species such as saithe, with species like whitting, haddock representative for the North–West subdivision, and the European plaice having the highest abundance in the South–East community^50^. The future fish stock and spatial distribution is however uncertain due to impacts of climate change related factors (e.g., growing temperatures)^49^ and overexploitation.

The most prominent human activities in the North Sea basin are fishing, coastal construction, maritime transport, oil and gas exploration and production, tourism, military, and OWF construction^38^. Within this list, the construction of OWFs has seen a rapid increase, aiming to reach a total cumulative installed capacity of 61.8–66.8 GW by 2030^51^. As indicated in Fig. 6, the new designated/search/scoping areas for the location of future OWFs will significantly increase the current space reserved for the offshore production of renewable energy in the North Sea basin.

### Spatio-temporal database of OWF developments in the North Sea basin

For the input of the geo-spatial layers with the location of OWF areas we compiled a comprehensive spatial data repository in QGIS containing the shapefiles of analysed OWF, from 1999 to 2027 (last year of available official information on OWF development, Appendix D). The analysis was performed for the North Sea geographic area, referred here as the basin scale, taking into account the cumulative pressures from individual OWF projects (project scale). The main data sources for geospatial information for OWF, for the entire North Sea basin, are EMODnet (Human Activities data portal) and OSPAR, which were complemented by data on the country level, where needed; i.e. from Crown Estate Scotland (Energy infrastructure, Legal Agreements), Rijkswaterstaat for the Netherlands. From the available geo-spatial data for OWF, we selected the OWF in our area of study (Fig. 6) with the status of consent-authorised, authorised, pre-construction, under construction, or fully commissioned (operational). Therefore, planned OWF such as Vesterhavet Syd and Vesterhavet Nord, for which the start date of construction is still unknown, were not included in the analysis. Similarly, for the Horns Rev 3 OWF no geo-referenced spatial footprint was available in the open-access data sets, and therefore it was not included in the analysis.

The collected OWF geospatial data was aggregated to create a geospatial database, for the studied period of 1999–2050, composed by the following attributes: code name, country, name, production capacity (MW), area (${\mathrm{km}}^{2}$), number of turbines, start operation (year), installation time, and status in the period 1999–2050 (construction, operation, decommissioning). The created geospatial dataset was additionally cross-checked for integrity with the information provided through the online platform 4coffshore.com.

The lack of data regarding the construction time was complemented with the methodology proposed by Lacal-Arántegui et al.^36^. Based on this research, we calculated the time required for OWF construction phase related activities multiplying 1.06 days by the known production capacity (total MW) for each analysed OWF.

The average time of operation is considered to be 20 years, probably profitably extendable to 25 years, as stated in a number of studies on the cycle of offshore wind farms^52^. For this case study, the operation time considered is 20 years (subject to change). Since there is little experience with the decommissioning of offshore wind farms (only a few OWFs have so far been decommissioned in the UK and Denmark), the decommissioning time is not yet clear. There are a number of parameters that influence the decommissioning time, which are: the number of turbines, the foundation type, the distance to port, etc. It is estimated that the time taken for decommissioning should be around 50–60% less than the installation time^37^. Our study considers the decommissioning time as 50% of the construction time.

### Time-aware cumulative effects assessment

In this study, Tools4MSP^53,54^, a Python-based Free and Open Source Software (FOSS) for geospatial analysis in support of Maritime Spatial Planning and marine environmental management, was used for the assessment of the impacts of OWFs on the marine ecosystem, in the three development stages. We applied the Tools4MSP CEA module to the OWF of the North Sea basin for the period 1999–2050, taking into account the full life cycle of the OWF development, namely the construction, operation and decommissioning phases. The modified methodology from Menegon et al.^31^ and subsequent implementation^55^, proposes to calculate the CEA score for each cell of analysis as follows (Eqs. 1, 2):

1 $$CEA=\sum_{k=1}^{n}d({E}_{k}) \sum_{j=1}^{m}{s}_{i,j} eff({P}_{j}{E}_{k})$$

where *eff* is the effect of pressure *P* over the environmental component *E* and is defined as follows:

2 $$eff \left({P}_{j}{E}_{k}\right)=(\sum_{i=1}^{l}{w}_{i,j} i({U}_{i},{M}_{i,j,k})){^{\prime}}$$

whereas,

- ${U}_{i}$ defines the human activity, namely the OWF activity in the study area
- ${E}_{k}$ defines the environmental components of the study area described in the Table 1
- ${d(E}_{k})$ defines intensity or presence/absence of the k-th environmental component
- ${P}_{j}$ defines the pressures exerted by human activities dependent on the three different OWF development phases (Annex B)
- ${w}_{i,j}$ refers to the specific pressure weight according to the OWF phase
- ${s(P}_{j}, {E}_{k}$) is the sensitivity of the k-th environmental component to the j-th pressure
- ${i({U}_{i, }M({U}_{i, }P}_{j}, {E}_{k}$)) is the distance model propagating j-th pressure caused by i-th activity over the k-th environmental component
- ${M(U}_{i}, {P}_{j}$) is the 2D Gaussian kernel function used for convolution, which considers buffer distances at 1 km, 5 km, 10 km, 20 km, and 50 km^56^.

**Table 1:** Primary sources for the environmental component data sets.

Environmental feature category	Environmental feature	Unit	Primary source (raw data)
Seabed habitats (EUNIS)	A3—infralittoral rock and other hard substrata A4—circalittoral rock and other hard substrata A5—sublittoral sediment	Presence/absence	https://www.emodnet-seabedhabitats.eu/access-data/download-data/ Feature: Broad-scale seabed habitat map (EUSeaMap)—EUNIS/full-detail classifications (updated 1st July 2019)
Marine mammal	Harbor purpoise	Probability of densities (nr. of animals per ${\mathrm{km}}^{2}$)	Waggitt, James (2020), Data from: “Distribution maps of cetacean and seabird population in the North-East Atlantic”, V6, Dryad, Dataset, https://doi.org/10.5061/dryad.mw6m905sz. The environmental components used in the binomial and Poisson models are annual temperature/variance, breeding colony index, breeding cycle, water depth, fronts, land, regional temperature, and seabed roughness
Birds	Razorbill, fulmar, guillemot	Probability of densities (nr. of animals per ${\mathrm{km}}^{2}$)	SEAPOP program (http://www.seapop.no/en/distribution-status/ ) through the online data portal (https://www2.nina.no/seapop/seapophtml/). The statistical model uses count data and geographically-fixed explanatory variables (geographic position, water depth, distance to coast) to predict the densities of the different species
Fish	Haddock	CPUE/absence and individual counts	https://datras.ices.dk/WebServices/Webservices.aspx
	Sandeel	CPUE/absence and individual counts	https://datras.ices.dk/WebServices/Webservices.aspx
	Whiting	CPUE/absence and individual counts	https://datras.ices.dk/WebServices/Webservices.aspx
	Saithe pollack	CPUE/absence and individual counts	https://datras.ices.dk/WebServices/Webservices.aspx
	Sprat	CPUE/absence and individual counts	https://datras.ices.dk/WebServices/Webservices.aspx

In Eq. (3), the CEA 1999–2050 describes the modelling over the time frame 1999–2050, whereas ${CEA}_{t}$ is the cumulative effect of year *t* within the timeframe 1999–2050:

$${CEA}_{1999-2050}= \sum_{t=1999}^{2050}{CEA}_{t}$$

In this study, each final CEA score was normalised. To normalise the value of each initial CEA score obtained using the Eq. (1), we calculated its percentage of the sum of all CEA scores for all OWFs in the three development phases, period spanning the period 1999–2050 ($${CEA}_{1999-2050})$$.

### Environmental components

The selection of the environmental components (receptors) impacted by the identified pressures is an essential part of the scoping phase for OWF location, as monitoring the status (distribution, abundance) of different identified species represents a relevant indicator for the ecosystem status. For the evaluation of the habitats and species that can be affected by the cumulative ecological effects of OWF, we adapted the methodology of Meissl et al.^14^. Therefore, we selected the environmental components based first on their: (1) ecological value, supported by legal documents identifying species protected by law or through various national and international agreements (e.g. EU Habitats Directive, Wild Mammals (Protection) Act (UK), see Table 1 in Appendix E), to which we added species with (2) commercial value, but also with a (3) broad geographic-scale habitat occurrence of the species in the studied area, based on previous studies^35^ and on 35 EIA studies for OWF in the North Sea basin.

Among the five fish species selected, sprat and sandeel play key roles in the marine food web (small pelagic fish), as prey source for piscivorous fish, cetacean and birds. The ecological value of sandeel, sprat, whiting and saither is also highlighted through EU or national protection agreements such as Priority Marine Features—PMF or Scottish/UK Biodiversity list (see Appendix E, Table 2). The list is completed by haddock, one of the fish species with commercial importance, highly dominant in the Central North Sea. With regards to the spatial occurrence at the basin level, the fish species selected are representative for both of the two distinct North Sea communities^50^, the southern part of the North Sea (sprat), and the northern and north-west part (haddock, whiting, saithe).

The three selected seabird species are of ecological importance for the marine ecosystem, as indicated through the European, national and international protection agreements, such as the EU Birds Directive Migratory Species or the IUCN Red List (see Appendix E, Table 1). While razorbill and guillemot have similar feeding and flying patterns (low flight, catch pray underwater), there is evidence of different behaviors towards OWFs, with relatively more avoidance from razorbill compared to guillemot. In relation to the spatial distribution of the three selected species, there is a clear distinction between razorbill, highly present in the coastal areas of west North Sea basin, guillemot, with a relatively even distribution across the marine basin, and fulmar, one of the 4 most common seabirds in the studied area, in particular in the central and N–E parts.

In the marine mammals category we selected the harbor porpoise, indicated to be one of the most impacted species in this category^57^, with a high occurrence in the North Sea basin. Its ecological value is emphasized by its presence in European and international lists for habitat protection, such as EU Habitats Directive^58^, OSPAR List of Threatened and/or Declining Species^59^, the Agreement on the Conservation of Small Cetaceans in the Baltic and the North Seas (ASCOBANS)^60^. The harbor porpoise is the protected species in numerous Natura 2000 areas in the North Sea basin, such as the Spatial Area of Conservation Southern North Sea^61^ (British EEZ) or The Special area of Protection Kleverbank^62^ (Dutch EEZ).

Among the selected fish species, sandeel had the highest occurrence in EIA studies of OWF developments (23 out of 35), while guillemot had the highest occurrence among seabird species (25 out of 35). With an occurrence of 26 out of the 35 analysed EIA document, the harbour porpoise is the most studied mammal in relation to the impact of OWF.

As a result, we selected three EUNIS marine seabed habitat types (European Union Nature Information System)^58^ (Appendix E, Table 2), three seabird species, one mammal species and five fish species (Appendix E, Table 1). The list can be extended; however, for this exercise we considered it sufficient.

The data sets used to represent the spatial distribution (presence/absence, intensity) of the environmental components in the studied area were obtained from multiple sources and were used in the Tools4MSP model either directly (EUNIS habitats, marine mammals, seabirds) or further processed using a predictive distribution model (fish species). In the case of EUNIS marine habitats, the data source was the online geo-portal EMODnet, through the Seabed Habitat service (Table 1), which provided GIS polygon layers for each habitat type and was further used to indicate presence/absence of a specific habitat.

For the distribution of the selected mammal species, the harbour porpoise, we used the modelling results of Waggit et al.^16^, translated into maps for the prediction of densities (nr. animals/${\mathrm{km}}^{2}$). The mapping approach starts with collating data from available surveys, which are further standardised with regards to transect length, number of platform sides, and the effective strip width. Finally, the standardised data sets were used in a binomial and a Poisson model, in association with environmental conditions (Table 1), in order to deliver a homogenous cover of species distribution maps, on 10 km × 10 km spatial resolution grid^16^.

For the distribution of the selected seabird species (razorbill, fulmar, guillemot), we used the results of the SEAPOP program (http://www.seapop.no/en/distribution-status/), through the open-source data portal (https://www2.nina.no/seapop/seapophtml/). The proposed methodology for creating the occurrence density prediction maps, on a 10 × 10 km spatial resolution grid, starts with the modelling of the presence/absence of birds using a binomial distribution and “logit link”. This was followed by the modelling of the number of birds using a Gamma distribution with a “log link” function, which also took into account geographically fixed explanatory variables (geographic position, water depth, and distance to coast).

The predictive model for the spatial distribution of fish species biomass (haddock, sandeel, whiting, saithe, sprat) was developed using AI4Blue software, an open-source, python-based library for Artificial Intelligence based geospatial analysis of Blue Growth settings (AI4Blue, 2021)^63^. The model was based on two types of inputs: (1) the observation data on the presence of species and (2) data on the absence of species (absence data) for the period 2000–2019. Both data types were extracted by the ICES North Sea International Bottom Trawl Survey (NSI-IBTS, extracted survey year 2000–2019 including all available quarters) for commercial fish species, which was accessed on the online ICES-DATRAS database^64^. Data was extracted using two DATRAS web service Application Programming Interfaces (APIs): (1) the *HHData*, that returns detailed haul-based meta-data of the survey (e.g. haul position, sampling method etc.) and (2) the *CPUEPerLengthPerHaulPerHour* for the catch/unit of effort per length of sampled species.

The presence data were represented by the catch/unit of effort (CPUE), expressed in kg of biomass of the specified species per one hour of hauling. The biomass was estimated by using the SAMLK (sex-maturity-age-length keys) dataset for ICES standard species. This approach is a viable alternative to presence-only data models, as it tackles the biased outcomes resulting from an non-uniform marine coverage of the data sets (mainly along the shipping routes)^65^. The absence data were estimated using the methodology presented by Coro et al.^65^, which detects absence location for the chosen species as the locations in which repeated surveys (with the selected species on the survey’s species target list) report information only on other species.

Additionally, the predictive model automatically correlates the presence/absence data with environmental conditions (Appendix E, Table 3) data to more accurately estimate the likelihood of species presence in the North Sea basin. Intersecting a large number of surveys containing observation data on the presence of selected species can return the true absence data locations, which represent a valuable indicator for geographical areas with unsuitable habitat (see methodology by Coro et al.^65^). Those locations were estimated from abiotic and biotic parameters and differed to the sampling absences which were estimated from surveys without presence data^65^. The environmental conditions (Appendix E, Table 2) data were accessed through direct queries using the MOTU Client option from the Marine Copernicus database. In order to input the layers to the CEA calculation, the input layer for the biomass was transformed using log[x + 1] to avoid an over-dominance of extreme values and all datasets rescaled from 0 to 1 in order to allow direct comparison on a single, unit-less scale^55^.

The rescaled special distribution of biomass for the selected species are presented in Appendix F (Fig. a–j).

### OWF pressures and relative weights

**Figure 7:**
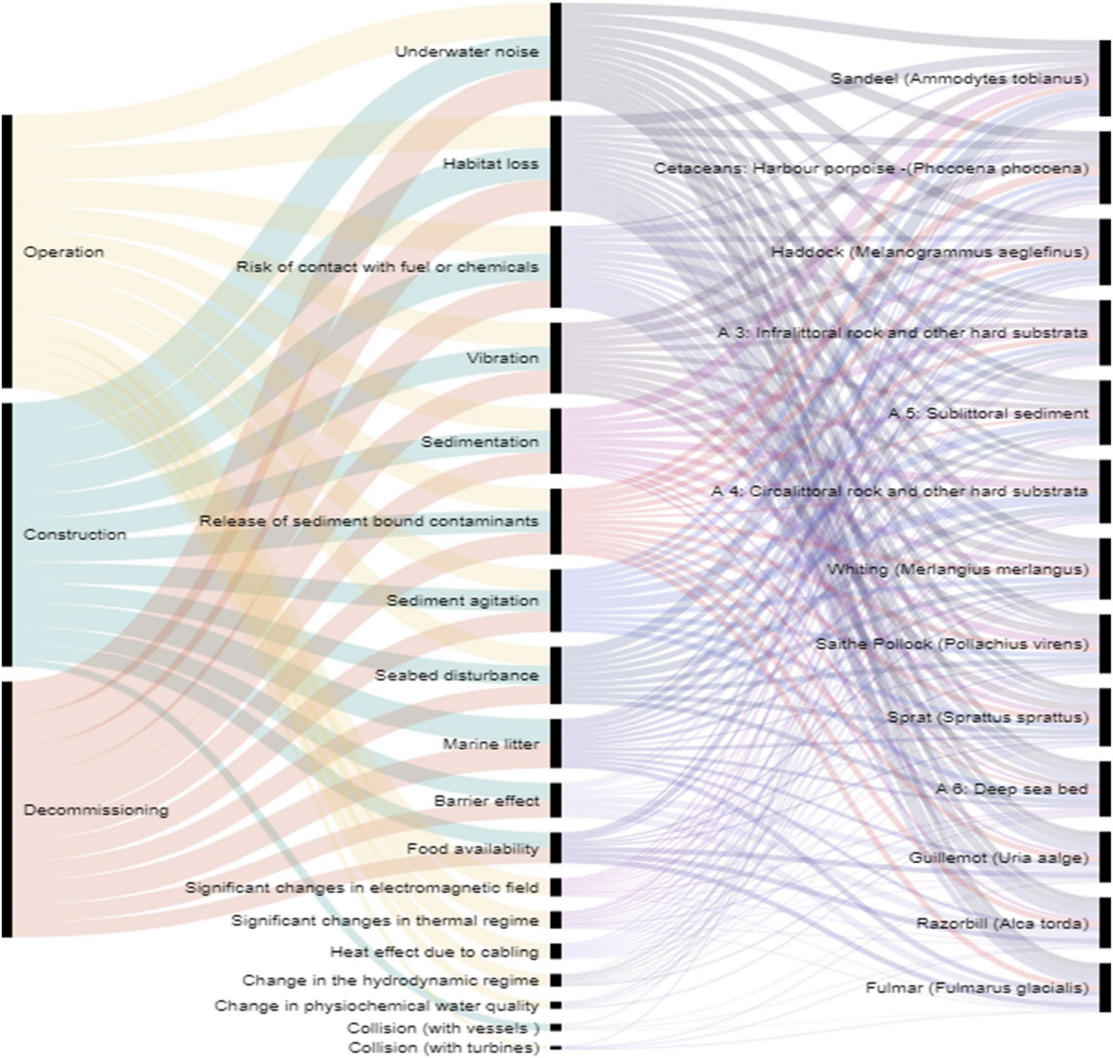
Impact chain defining OWF phases-pressure-environmental components analysed in the North Sea (the strength of the link between pressures and environmental components is proportional to the sensitivity scores. The order is descending from the pressures with highest impact, as well as from the environmental components most affected).

A systematic literature review was conducted to reach a first quantification of the OWF pressure weights ($${w}_{i,j}$$,) in the construction, operation, and decommissioning phases ($${U}_{i})$$. The OWF-related pressures specific to each of the phases of the OWF life cycle were based on the comprehensive analysis of all the existing Environmental Impact Assessment (EIA) methodologies used in the North Sea countries^14^. The review enabled the collection of 18 pressures that were subsequently compared and merged with the pressures established in the Marine Strategy Framework Directive, applied by the EU countries in the assessment of environmental impacts^66^. Figure 7 illustrates the impact chain linking the three OWF development phases with the exerted 18 pressures and the 12 selected environmental components impacted.

Sensitivity in this research is defined as the likelihood of change when a pressure is applied to a receptor (environmental component) and is a function of the ability of the receptor to adapt, tolerate or resist change and its ability to recover from the impact^67^. The criteria for assessing the sensitivities of environmental components is based on MarLIN (Marine Life Information Network) detailed criteria (https://www.marlin.ac.uk/sensitivity/sensitivity_rationale).

**Figure 8:**
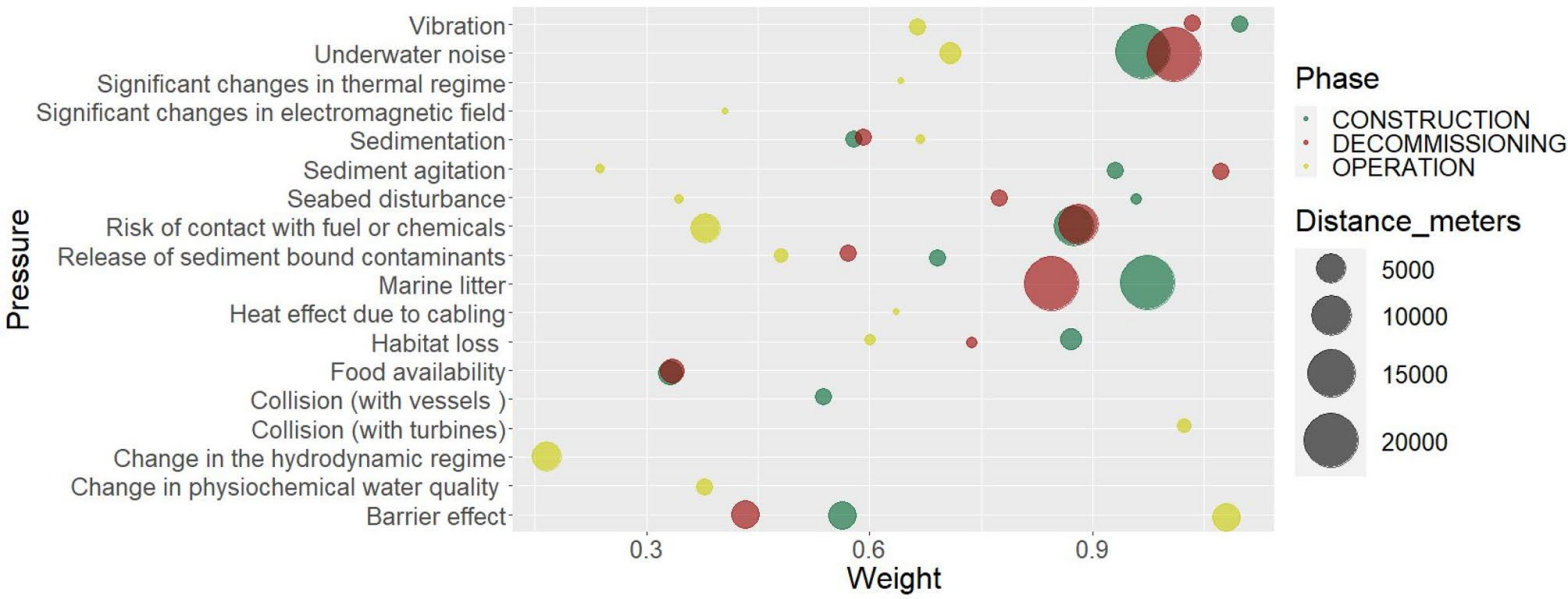
Pressure weights and pressure propagation distance in meters in each of the development phases of the offshore wind farms.

We validated the weights of pressures (${w}_{i,j}$ from 0 to 5) and scores of environmental components sensitivities (${s(P}_{j}, {E}_{k}$) from 0 to 5), as well as the distance of pressure propagation (≤1000 m to ≥ 25,000 m), through a series of 4 questionnaires for the marine mammals, seabirds, fish and seabed habitats. The compiled questionnaires were further validated through semi-interviews of 9 experts in the field of marine ecology, spatial planning, environmental impact assessment and offshore wind energy development. The expert-based questionnaires also included a confidence level for the proposed scores, which ranged between 0.2 (very low confidence: based on expert judgement; proxy assessment) and 1 (very high confidence: based on peer reviewed papers, report, assessment on the same receptor). The confidence level was used in determining the final scores for the pressure weights and species sensitivities. The final scores for weights and sensitivity scores were identified either by calculating the mean value (for cases where literature review scores and expert scores differed by > 2 units) or selecting the higher value—precautionary principle (for cases where scores from different sources differed by < 2 units). The definitions used, the values for the final environmental components sensitivity scores and the pressure weights are presented in Appendix G, Tables 9, 10, 11, 12, 13. Figure 8 illustrates the relative weights of the 18 analysed pressures in the three development phases. Here, a distinction can be made between localised pressures (e.g., Release of Sediment bound contaminants, sedimentation) and pressures with a higher spatial distribution (e.g., Underwater noise, Marine litter).

### Representation of the spatio-temporal distribution of CEA scores

The cumulative impacts of OWFs over the 1999–2050 timeline (Fig. 2b) were represented in hexagonal choropleth maps using the outputs of the Tools4MSP model, over the analysed timeline 1999–2050. The original output of the Tools4MSP model was resampled over a hexagonal grid with a cell size of 10 km resolution (which corresponds with the distance (d) between the centroids of neighbouring cells). The hexagonal cells representing the CEA scores were then grouped in five classes, for the three selected years: 2020 (current state), 2030 (related to the EU energy targets benchmark year), and 2046 (final part of the analysed period, with higher impacts of the decommissioning phase). The final maps were realised using the open source software QGis 3.6.0^68^.

The figures representing the temporal distribution of OWF areas, the CEA scores by phase (Fig. 1) and by country (Fig. 2a) were realised using Excel. The distribution of CEA scores by individual OWF, the link between pressures and environmental components, CEA scores per species (Fig. 4) and the pressure weights scatter plot (Fig. 8) were realised using R 4.0.2^69^. The alluvial diagram representing the impact chain between OWF phases, pressures and species was realised using RawGraphs (https://rawgraphs.io/).

### Sensitivity analysis

A sensitivity analysis was performed to understand the influence of the modeled parameters on the CEA results. For this purpose the Tools4MSP Software V3.0 (2021)^70^ implements a novel module that enables a sensitivity analysis, applicable to CEA and to any other modules of Tools4MSP Modeling framework. In this study, three sensitivity analyses were performed, one for each OWF phase (construction, operation and decommissioning). For each phase two groups of uncertainty components were analyzed: the pressure propagation model (distance and weight) for each pressure generated within each OWF development phase and the effect functions, which models the effects of the generated pressures on the environmental components. For each phase 5600 randomized model runs were performed and CEA results were used to estimate the Total Order Index for each input variables (effect functions and pressure propagations). The confidence level included in the expert-based questionnaire was used to model the variability of input variables (uncertainty components) of the randomized model runs. The Total Order Index expresses the contribution of each variable in determining the output variance (uncertainty) including all variance caused by its interactions with any other uncertainty component. The advantage of the Total Order Index is that it can measure the effect of interactions in non-additive systems^71^.

## Supplementary Information

Supplementary Information.

## Data Availability

All geo-spatial data used as initial inputs (raw data) in this study are available from open source repositories, as described in the Supplementary information files, namely Appendix E (marine species, environmental features) and Appendix A (offshore wind farms). The species sensitivity scores, relative importance weight and distance propagation of pressures, obtained through literature review and validated through interviews, and the CEA scores calculated through our methodology for each individual OWF, are available from the corresponding author upon reasonable request. The satellite image used in Figs. 2b, 3a, 6, A1, A2, A3, A4, A 5, Appendix C and Appendix F (figure j) was generated using the QuickMapServices plugin of QGIS 3.6.0 which retrieved the OSM Standard (open street map standard) from OpenStreetMap database^72^ (https://planet.openstreetmap.org). OpenStreetMap is an editable map database distributed over the Open Data Commons Open Database License^73^.
