# Supplementary material for: Spatial and temporal analysis of cumulative environmental effects of offshore wind farms in the North Sea basin
Source: Sci Rep. 2021 May 12;11:10125. doi: 10.1038/s41598-021-89537-1 (PMC8115305; doi:10.1038/s41598-021-89537-1)
Supplement: Supplementary file 1 — Supplementary Information. [file 41598_2021_89537_MOESM1_ESM.docx]

**Supplementary information**

**TITLE: “Spatial and temporal analysis of cumulative environmental effects of offshore wind farms in the North Sea basin”**

# AUTHORS: Laura Florentina Gusatu1, Stefano Menegon2, Daniel Depellegrin3, Christian Zuidema1, André Faaij4, Claudia Yamu1

**ORCID ID:**

Laura Florentina Gusatu: 0000-0003-0544-9416

Stefano Menegon: 0000-0003-4488-8745

Daniel Depellegrin: 0000-0002-6493-9506

Christian Zuidema: 0000-0001-5055-5053

André Faaij: 0000-0002-1224-5940

Claudia Yamu: 0000-0002-2847-4975

**AUTHOR ADDRESSES:**

1University of Groningen, Faculty of Spatial Sciences, Department of Planning, 9747 AD Groningen, the Netherlands

2CNR – National Research Council of Italy, ISMAR – Institute of Marine Sciences, Castello 2737/F, 30122 Venice, Italy

3Renewable Energy Group, College of Engineering, Mathematics and Physical Sciences, University of Exeter, Cornwall Campus, Penryn, United Kingdom

4University of Groningen, Faculty of Science and Engineering, 9747 AD Groningen, the Netherlands

**EMAIL:**

Laura Florentina Gusatu : [l.f.gusatu@rug.nl](mailto:l.f.gusatu@rug.nl)

Stefano Menegon: stefano.menegon@cnr.it

Daniel Depellegrin: D.D.Depellegrin@exeter.ac.uk

Christian Zuidema: c.zuidema@rug.nl

André Faaij: a.p.c.faaij@rug.nl

Claudia Yamu: claudia.yamu@rug.nl

**Correspondence to:** [l.f.gusatu@rug.nl](mailto:l.f.gusatu@rug.nl)

Appendix A: Below we have listed the spatial location and attributes of the analyzed OWF: country, name, area size, number of turbines, start construction, start operation date.

*Information collected from available open source data bases (EMODNET, OSPAR, Crown Estate Scotland, Rijskwaterstaat) complemented by data from 4coffshore.com and the methodology of Lacal-Arántegui et al. (2018).


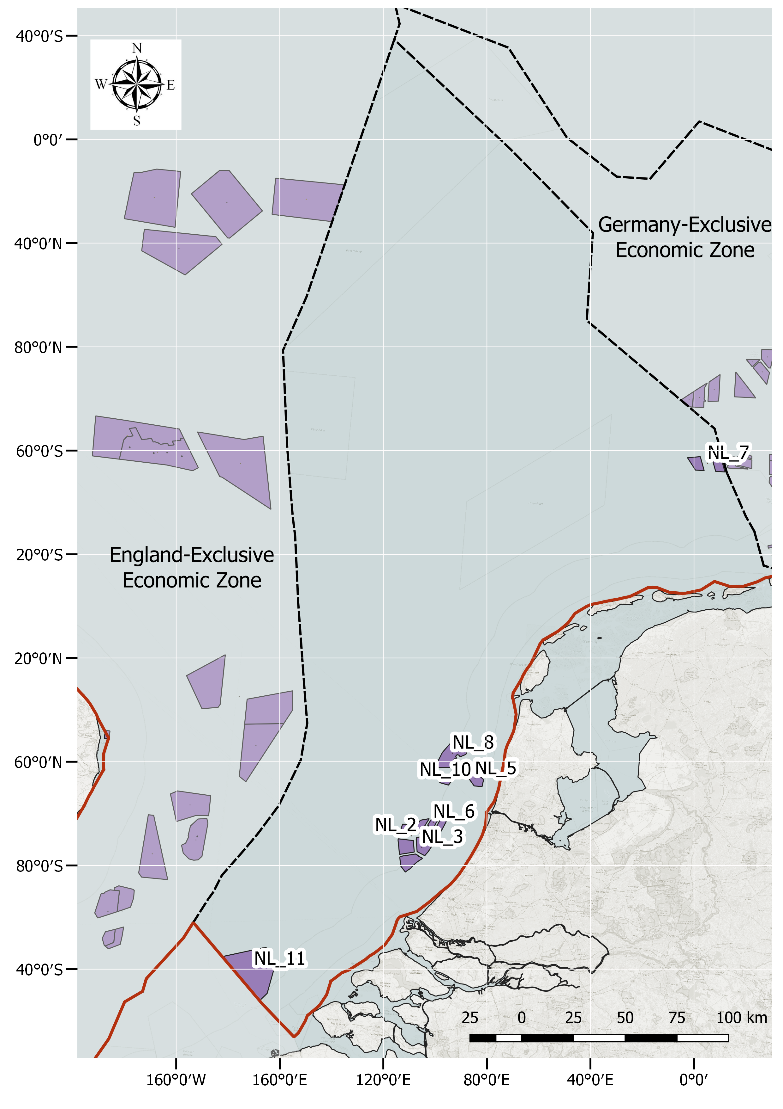


Fig. A1. OWF database – The Netherlands EEZ

Table 4. OWF database - The Netherlands EEZ

| ID | Country | Name | Production capacity (MW) | Area ($\boldsymbol{km}^{\boldsymbol{2}}\boldsymbol{)}$ | Number of turbines | Start operation year | Construction time (years ESTIMATION)* |
| --- | --- | --- | --- | --- | --- | --- | --- |
| NL_8 | The Netherlands | Nord-Holland boven Noordzeekanaal | 700 | 126.42 | 126 | 2024 | 1 |
| NL_3 | The Netherlands | Hollandese Kust III and IV | 770 | 108.17 | 70 | 2023 | 2 |
| NL_2 | The Netherlands | Hollandese Kust I and II | 770 | 103.06 | 70 | 2022 | 2 |
| NL_11 | The Netherlands | Borssele 1, 2, 3, 4 | 1483.5 | 344.92 | 171 | 2021 | 3 |
| NL_7 | The Netherlands | Gemini | 600 | 67.62 | 150 | 2016 | 1 |
| NL_6 | The Netherlands | Eneco Luchterduinen | 129 | 15.9 | 43 | 2015 | 1 |
| NL_10 | The Netherlands | Prinses Amaliawindpark | 120 | 16.73 | 60 | 2007 | 1 |
| NL_5 | The Netherlands | Egmond aan Zee | 108 | 24.46 | 36 | 2007 | 1 |


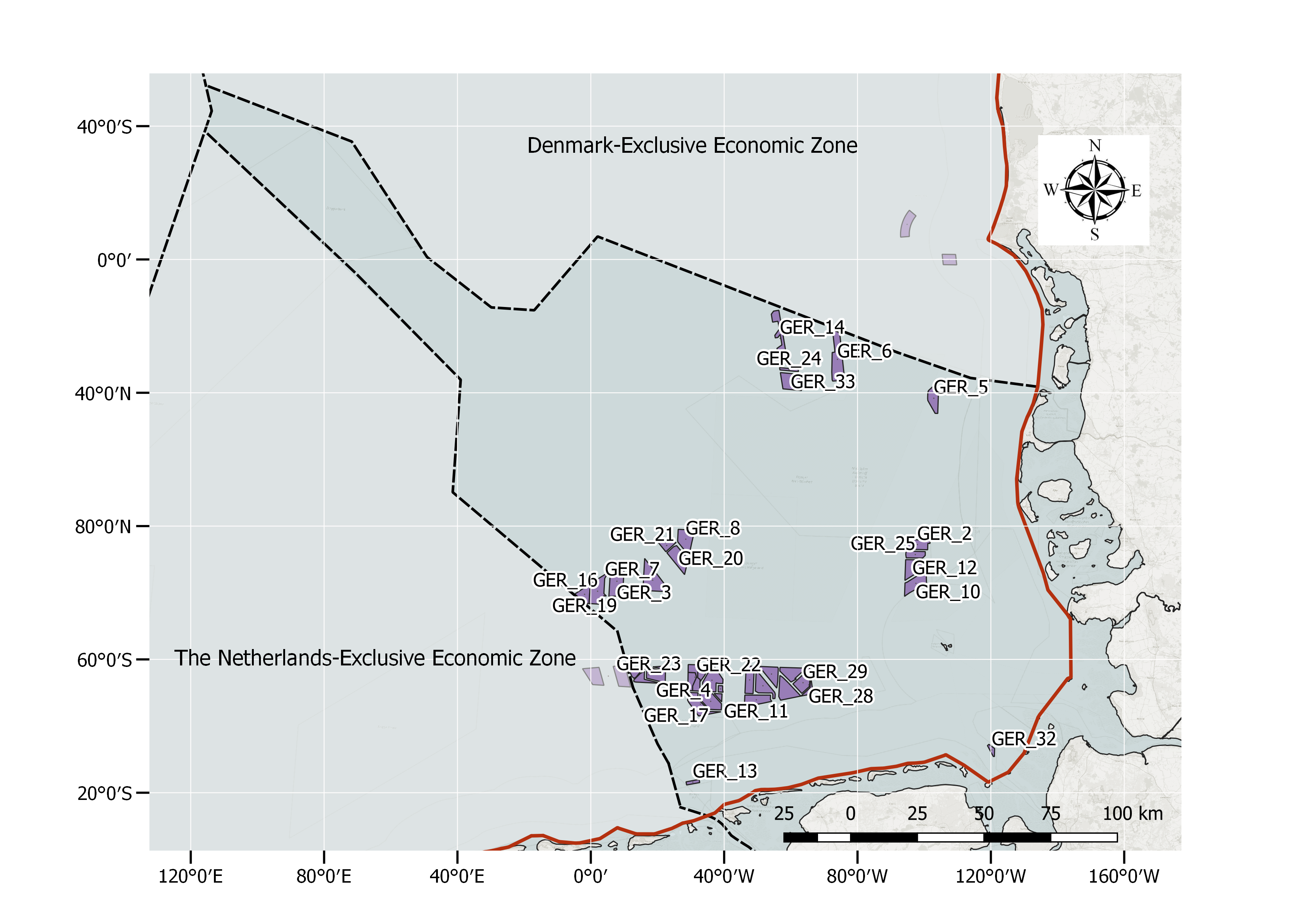


Fig. A2. OWF database – German EEZ

Table 5. OWF database– German EEZ

| ID | COUNTRY | NAME | PRODUCTION CAPACITY (MW) | | | AREA ($\boldsymbol{km}^{\mathbf{2}}\mathbf{)}$ | NUMBER OF TURBINES | START OPERATION YEAR | CONSTRUCTION TIME (YEARS ESTIMATION)* |
| --- | --- | --- | --- | --- | --- | --- | --- | --- | --- |
| GER_31 | Germany | N-3.5 | | 420 | 33.6 | | 2 | 2028 | 1 |
| GER_27 | Germany | N-3.6 | | 480 | 31.16 | | 2 | 2028 | 1 |
| GER_30 | Germany | N-3.8 | | 433 | 29.04 | | 2 | 2026 | 1 |
| GER_29 | Germany | N_3.7 | | 225 | 29.3 | | 2 | 2026 | 1 |
| GER_28 | Germany | Gode Wind 3 | | 241.75 | 11.62 | | 18 | 2024 | 1 |
| GER_7 | Germany | EnBW He Dreiht | | 900 | 62.5 | | 90 | 2025 | 3 |
| GER_23 | Germany | Borkum Riffgrund 3 | | 900 | 60.12 | | 81 | 2024 | 2 |
| GER_25 | Germany | Kaskasi | | 325 | 17.55 | | 38 | 2022 | 1 |
| GER_21 | Germany | Albatros | | 112 | 12.02 | | 16 | 2020 | 1 |
| GER_22 | Germany | Trianel Borkum II | | 202.56 | 33.07 | | 32 | 2019 | 1 |
| GER_20 | Germany | Hohe See | | 497 | 40.43 | | 71 | 2019 | 1 |
| GER_19 | Germany | Deutsche Bucht | | 269 | 22.59 | | 31 | 2019 | 1 |
| GER_18 | Germany | Merkur | | 396 | 46.85 | | 66 | 2018 | 1 |
| GER_17 | Germany | Borkum Riffgrund 2 | | 450 | 44.59 | | 56 | 2018 | 1 |
| GER_16 | Germany | Veja Mate | | 402 | 50.72 | | 67 | 2017 | 1 |
| GER_33 | Germany | Noerdlicher grund | | 384 | 41.83 | | 64 | 2017 | 1 |
| GER_32 | Germany | Nordergr?nde | | 110.7 | 5.88 | | 18 | 2017 | 1 |
| GER_11 | Germany | Nordsee One | | 332 | 33.53 | | 54 | 2017 | 2 |
| GER_14 | Germany | Sandbank | | 288 | 44.42 | | 72 | 2016 | 1 |
| GER_9 | Germany | Gode Wind 1 and 2 | | 582 | 69.54 | | 97 | 2016 | 1 |
| GER_5 | Germany | Butendiek | | 288 | 31.45 | | 80 | 2015 | 1 |
| GER_24 | Germany | Noerdlicher Grund Teil Sandbank | | 80 | 6.08 | | 16 | 2015 | 1 |
| GER_4 | Germany | Borkum Riffgrund 1 | | 312 | 35.71 | | 78 | 2015 | 2 |
| GER_2 | Germany | Amrumbank West | | 302 | 30.18 | | 80 | 2015 | 2 |
| GER_12 | Germany | Nordsee Ost | | 295.2 | 35.35 | | 48 | 2014 | 2 |
| GER_13 | Germany | Riffgat | | 108 | 5.99 | | 30 | 2014 | 2 |
| GER_6 | Germany | DanTysk | | 288 | 64.53 | | 80 | 2014 | 2 |
| GER_8 | Germany | Global Tech I | | 400 | 39.99 | | 80 | 2014 | 2 |
| GER_10 | Germany | Meerwind Sud/Ost | | 288 | 39.78 | | 80 | 2014 | 2 |
| GER_15 | Germany | Trianel Borkum I | | 200 | 22.6 | | 40 | 2015 | 4 |
| GER_3 | Germany | Bard Offshore 1 | | 400 | 56.78 | | 80 | 2011 | 1 |
| GER_1 | Germany | Alpha Ventus | | 60 | 3.99 | | 12 | 2009 | 1 |


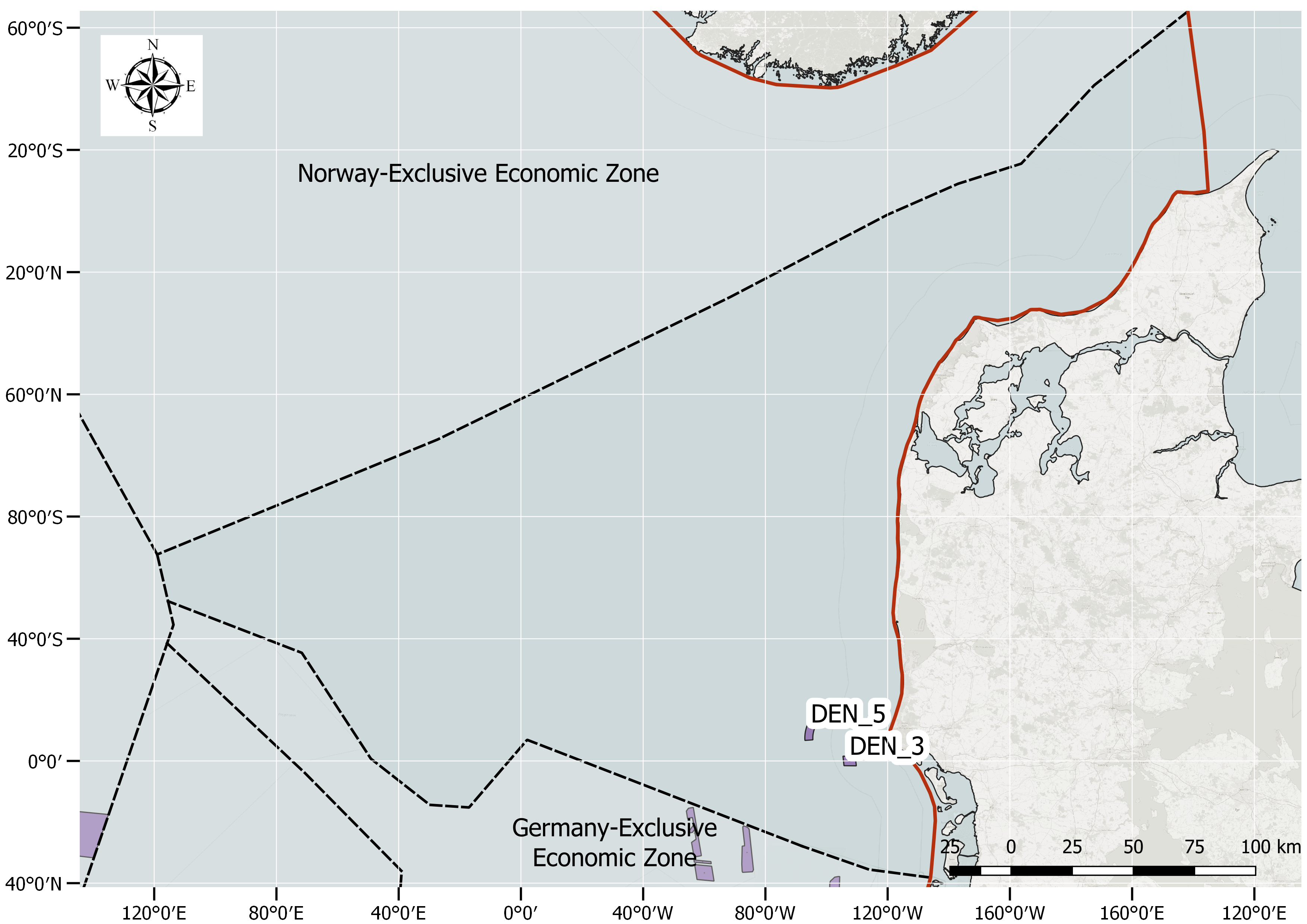


Fig. A3. OWF database – Denmark EEZ

Table 6. OWF database– Denmark EEZ

| ID | COUNTRY | NAME | PRODUCTION CAPACITY (MW) | AREA ($\boldsymbol{km}^{\mathbf{2}}\mathbf{)}$ | NUMBER OF TURBINES | START OPERATION YEAR | CONSTRUCTION TIME (YEARS ESTIMATION)* |
| --- | --- | --- | --- | --- | --- | --- | --- |
| DEN_5 | Denmark | Horns Rev 2 | 209 | 31.38 | 91 | 2009 | 1 |
| DEN_3 | Denmark | Horns Rev 1 | 160 | 19.62 | 80 | 2002 | 1 |


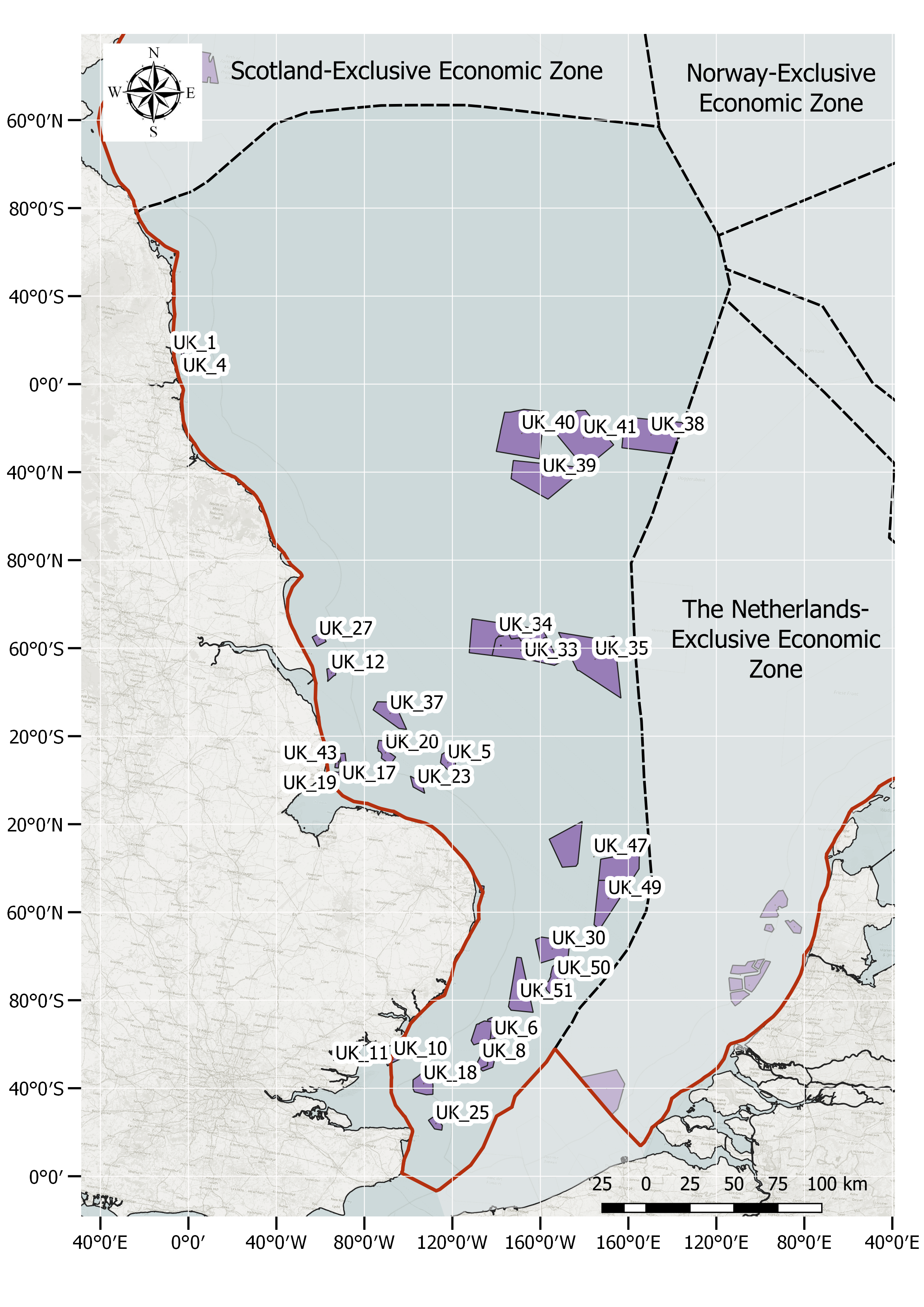


Fig. A4. OWF database – England EEZ

Table 7. OWF database– England EEZ

| ID | COUNTRY | NAME | PRODUCTION CAPACITY (MW) | AREA ($\boldsymbol{km}^{\mathbf{2}}\mathbf{)}$ | NUMBER OF TURBINES | START OPERATION YEAR | CONSTRUCTION TIME (YEARS ESTIMATION)* |
| --- | --- | --- | --- | --- | --- | --- | --- |
| UK_30 | England | East Anglia ONE North | 800 | 208.09 | 67 | 2027 | 1 |
| UK_51 | England | East Anglia TWO | 900 | 255.22 | 75 | 2027 | 2 |
| UK_34 | England | Hornsea project two | 1386 | 461.89 | 165 | 2022 | 2 |
| UK_38 | England | Dogger Bank - Teesside A | 1200 | 560.17 | 120 | 2025 | 3 |
| UK_49 | England | East Anglia THREE | 1400 | 305.89 | 172 | 2026 | 4 |
| UK_40 | England | Dogger bank Creyke beck B | 1200 | 599.51 | 100 | 2024 | 3 |
| UK_47 | England | Norfolk Vanguard | 1800 | 592.37 | 180 | 2026 | 5 |
| UK_33 | England | Hornsea project one | 1218 | 407.19 | 174 | 2019 | 1 |
| UK_35 | England | Hornsea project three | 2400 | 696.77 | 300 | 2025 | 4 |
| UK_37 | England | Triton Knoll | 860 | 149.48 | 90 | 2021 | 1 |

Table 7. OWF database– England EEZ (continued)

| ID | COUNTRY | NAME | PRODUCTION CAPACITY (MW) | AREA ($\boldsymbol{km}^{\mathbf{2}}\mathbf{)}$ | NUMBER OF TURBINES | START OPERATION YEAR | CONSTRUCTION TIME (YEARS ESTIMATION)* |
| --- | --- | --- | --- | --- | --- | --- | --- |
| UK_39 | England | Dogger Bank - Creyke A | 1200 | 515.29 | 100 | 2023 | 3 |
| UK_41 | England | Sofia | 1400 | 593.77 | 140 | 2024 | 4 |
| UK_4 | England | Blyth Offshore Demonstrator Project - Array 2 | 41.5 | 6.98 | 5 | 2018 | 1 |
| UK_50 | England | East Anglia ONE | 714 | 162.82 | 102 | 2019 | 2 |
| UK_5 | England | Dudgeon | 402 | 55.16 | 67 | 2017 | 1 |
| UK_6 | England | Galloper | 353 | 113.65 | 56 | 2017 | 1 |
| UK_20 | England | Race Bank | 573.3 | 62.38 | 91 | 2017 | 1 |
| UK_27 | England | Westermost Rough | 210 | 34.93 | 35 | 2015 | 1 |
| UK_12 | England | Humber Gateway | 219 | 26.54 | 73 | 2015 | 2 |
| UK_11 | England | Gunfleet Sands 3 - Demonstration Project | 12 | 2.55 | 2 | 2013 | 1 |
| UK_17 | England | Lincs | 270 | 38.31 | 75 | 2012 | 1 |
| UK_18 | England | London Array | 630 | 122.48 | 175 | 2012 | 1 |
| UK_25 | England | Thanet | 300 | 34.97 | 100 | 2010 | 1 |
| UK_23 | England | Sheringham Shoal | 316.8 | 34.99 | 88 | 2011 | 2 |
| UK_8 | England | Greater Gabbard | 504 | 146.24 | 140 | 2010 | 1 |
| UK_10 | England | Gunfleet Sands | 172 | 15.81 | 48 | 2009 | 1 |
| UK_43 | England | Inner Dowsing | 97.2 | 8.81 | 27 | 2008 | 1 |
| UK_19 | England | Lynn | 97.2 | 7.88 | 27 | 2008 | 1 |
| UK_22 | England | Scroby Sands | 60 | 8.91 | 30 | 2004 | 1 |
| UK_1 | England | Blyth | 4 | 0.4 | 2 | 2000 | 1 |


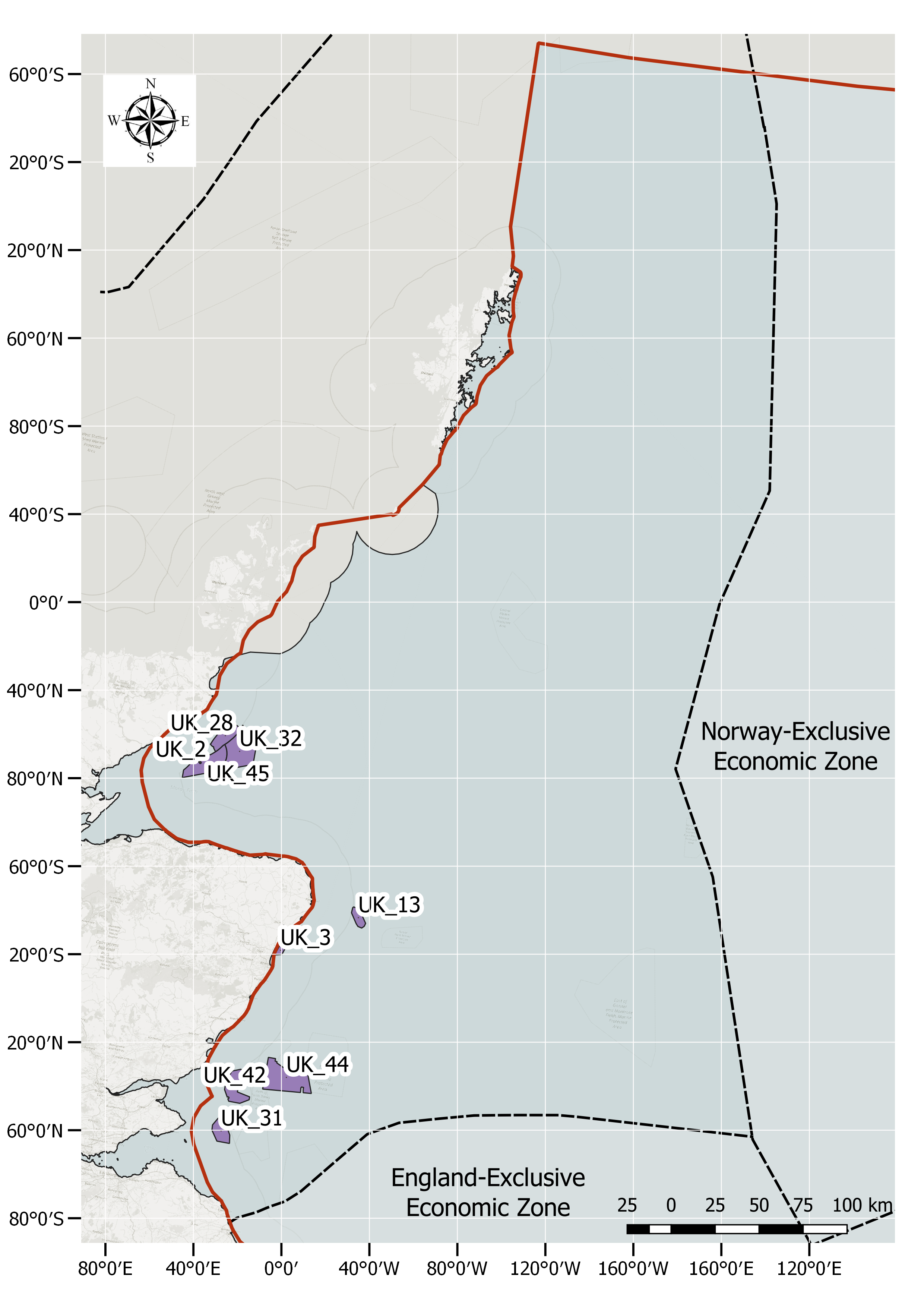


Fig. A5. OWF database – Scotland EEZ

Table 8. OWF database– Scotland EEZ

| ID | COUNTRY | NAME | PRODUCTION CAPACITY (MW) | AREA ($\boldsymbol{km}^{\mathbf{2}}\mathbf{)}$ | NUMBER OF TURBINES | START OPERATION YEAR | CONSTRUCTION TIME (ESTIMATED YEARS)* |
| --- | --- | --- | --- | --- | --- | --- | --- |
| UK_45 | Scotland | Moray West | 850 | 225.32 | 85 | 2026 | 2 |
| UK_44 | Scotland | Seagreen - Phase One | 1075 | 391.16 | 114 | 2024 | 3 |
| UK_31 | Scotland | Neart na Gaoithe | 448 | 105.17 | 54 | 2022 | 2 |
| UK_42 | Scotland | Inch Cape | 784 | 150 | 72 | 2022 | 2 |
| UK_32 | Scotland | Moray East | 950 | 295.5 | 100 | 2021 | 2 |
| UK_28 | Scotland | Beatrice | 588 | 131.52 | 84 | 2018 | 1 |
| UK_13 | Scotland | Hywind Scotland Pilot Park | 30 | 53.77 | 5 | 2018 | 1 |
| UK_3 | Scotland | Aberdeen Offshore Wind Farm (EOWDC) | 93.2 | 20.01 | 11 | 2018 | 2 |
| UK_2 | Scotland | Beatrice Demonstration | 10 | 1.46 | 2 | 2007 | 1 |

Appendix B. Sensitivity analysis of effect functions and pressure propagations defined as total order index (in %) for each OWE phase (a – construction, b – operational and c - decommissioning). The Total Order Index expresses the contribution of each variable in determining the output variance (uncertainty) including all variance caused by its interactions, of any order, with any other input variables.


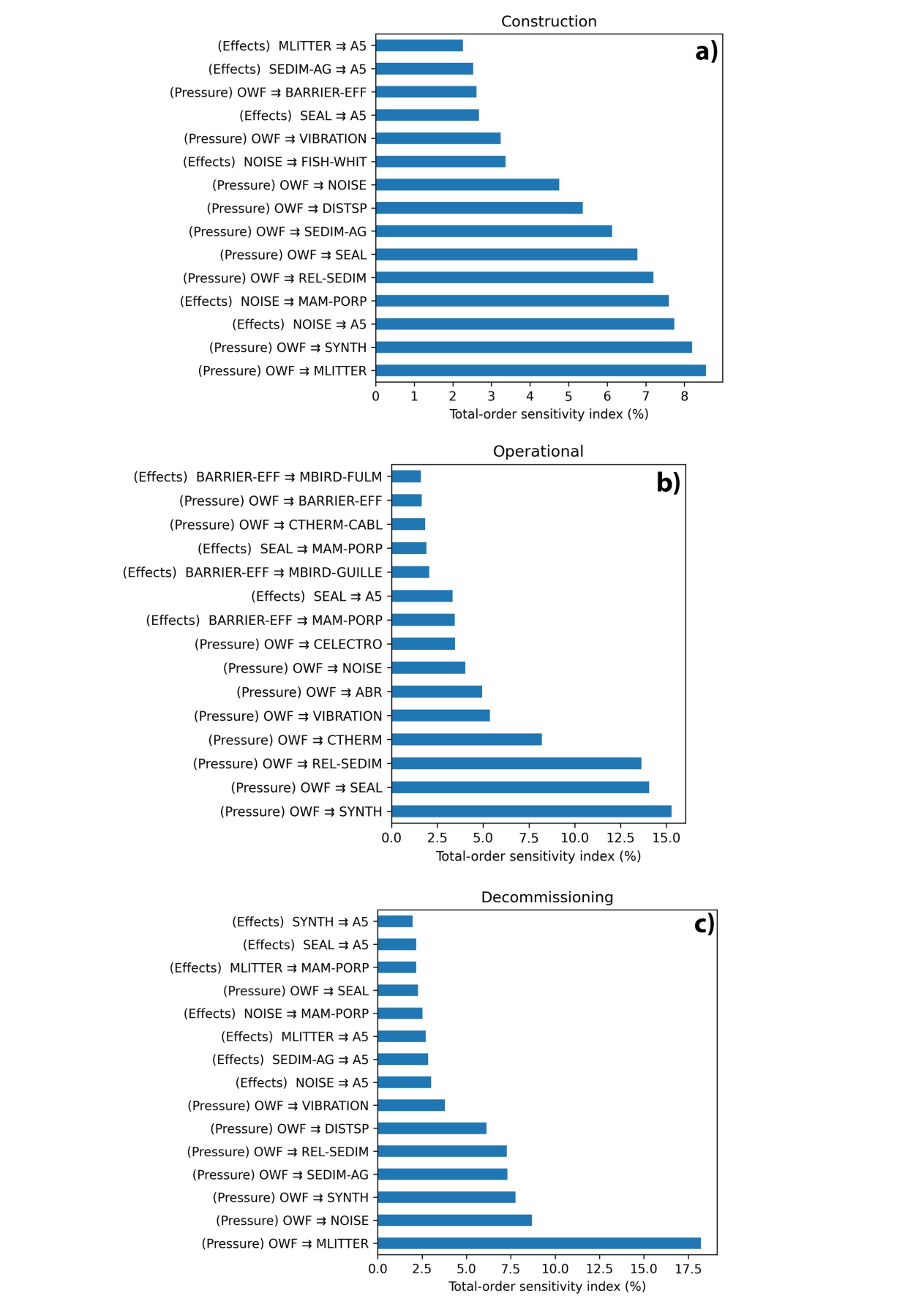


Appendix C: Offshore wind farm areas and fishing intensity in the North Sea (beam trawls and bottom otter trawls)


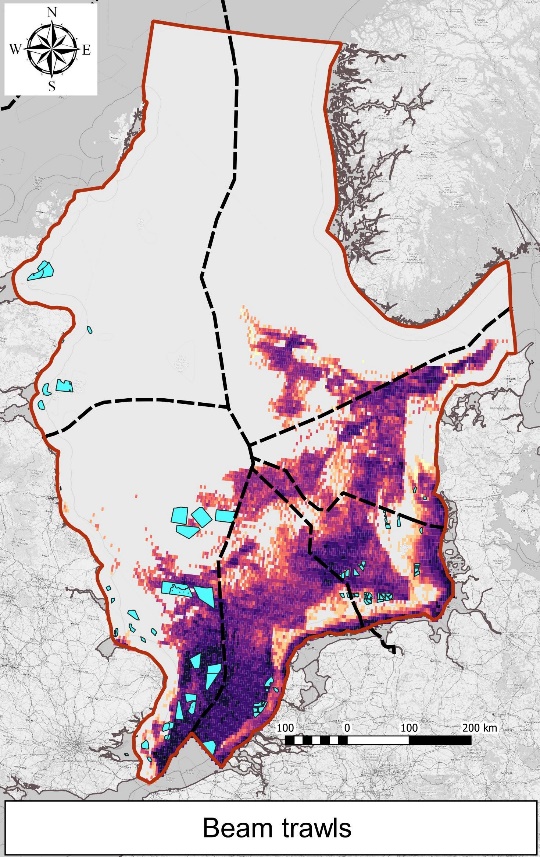

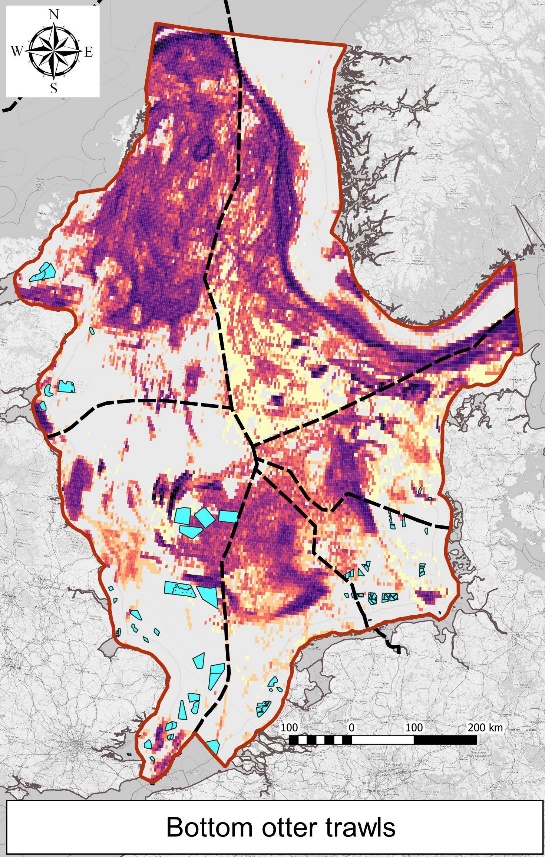

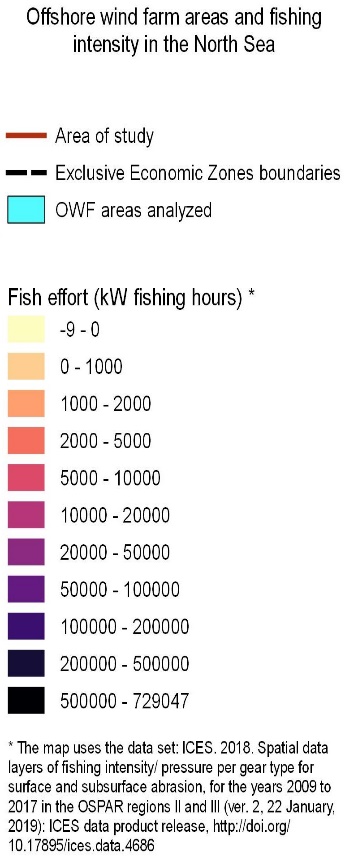


Appendix D: Timeline of all the analyzed offshore wind farms in the North Sea, incorporating for each of the analyzed OWF the entire lifecycle: construction, operation, decommissioning. The chronological representation of the OWF life cycle in the analyzed area indicates an alternation in the deployment of wind farms by country, over the period 1999-2050.


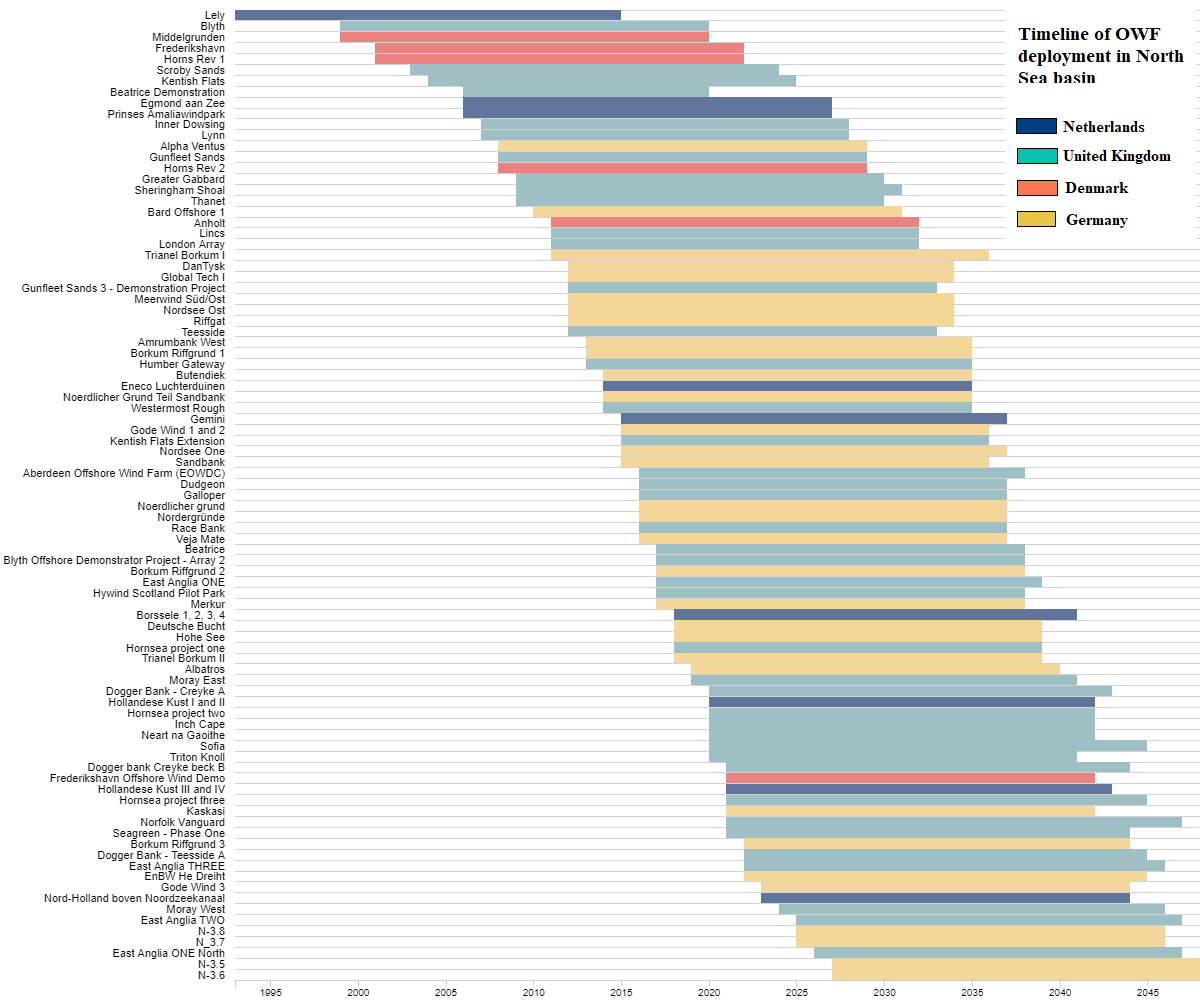


Appendix E: The list of selected environmental components and environmental features used in predictive model, with the specification of their sources.

Table 1. Selected mammals, birds and fish species

| Species | Distribution / occurrence | Protected by EU Directives / other agreements / commercial importance |
| --- | --- | --- |
| Harbour porpoises (Phocoena phocoena) | Predicted to occur in high densities in the area, in particular in the western and central North Sea^35^. Indicated to be the highest impacted species by anthropogenic pressures among the mammals category^57^ . | EU Habitats Directive^58^ / OSPAR List of Threatened and/or Declining species^59^ / Agreement on the Conservation of Small Cetaceans in the Baltic and the North Seas (ASCOBANS)^60^ /Wild Mammals (Protection) Act (UK)^72^ /UK Biodiversity Action Plan (Priority Species)^73^. |
| Razorbill  (Alca torda) | Prevalent mainly in the proximity of the coastal areas of the S-E, S and S-W North Sea ^35^ . | BoCC Amber listed^74^, Birds Directive Migratory Species^75^, IUCN Red List^76^ ‘Near Threatened’ status |
| Fulmar  (Fulmarus glacialis) | One of the 4 most common seabirds in the North Sea^77^ | BoCC Amber listed^74^, Birds Directive Migratory Species^75^, IUCN Red List^76^ ‘Least Concern’ status |
| Guillemot  (Uria aalge) | One of the 4 most common seabirds in the North Sea^77^ | BoCC Amber listed^74^, EU Birds Directive Migratory Species^75^, IUCN Red List^76^ ‘Least Concern’ status |
| Haddock (Melanogrammus aeglefinus) | Most predominant fish species in the Central North Sea fish community (42,4%) and second most predominant specie in the North Sea shelf edge fish community (11,6%) ^34^ | Species of commercial importance |
| Sandeel  (Ammodytes tobianus) | Constitutes a large proportion of the fish biomass in the North Sea^35^ and is part of the major prey for fish, seabirds^78^ and marine mammals ^79^ | Scottish Nature Conservation Marine Protected Area^80^ search feature / Priority Marine Features - PMF ^81^(Scotland)  Species of commercial importance |
| Whiting  (Merlangius merlangus) | Second most predominant fish species in the Eastern North Sea fish community^34^ and is part of the major prey for marine mammals ^79^ | Scottish Nature Conservation Marine Protected Area (MPA)^80^ search feature (juvenile) / Scottish Biodiversity List^82^ / UK Biodiversity Action Plan ^73^(UK BAP) / Priority Marine Features - PMF^81^ (Scotland) juveniles |
| Saithe Pollock (Pollachius virens) | The predominant species in the North Sea shelf edge fish community (43.6%)^34^. | Priority Marine Features - PMF^81^ (Scotland) |
| Sprat  (Sprattus sprattus) | Predominantly located in the central and southern parts of the North Sea^83^. | Priority Marine Features - PMF^81^ (Scotland) / UK Biodiversity Action Plan^73^ (UK BAP). |

Table 2. Selected EUNIS marine seabed habitats

| EUNIS benthic habitats^58^ | *EUNIS habitat code* | *EUNIS habitat name* | *Description (EUNIS habitat classification 2007)* |
| --- | --- | --- | --- |
|  | A3 | A3 :Infralittoral rock and other hard substrata | Includes habitats of bedrock, boulders and cobbles which occur in the shallow subtidal zone and typically support seaweed communities^84^ |
|  | A4 | A4: Circalittoral rock and other hard substrata | Characterized by animal dominated communities, which vary and is impacted by wave action, tidal stream strength, salinity, turbidity, the degree of scouring and rock topography^84^ |
|  | A5 | A5: Sublittoral sediment | Sediment ranges from boulders and cobbles, through pebbles and shingle, coarse sands, sands, fine sands, muds, and mixed sediments^84^ |

Table 3. Environmental features for the species predictive distribution model

| Environmental feature name | Service ID : source / Marine Copernicus service (https://resources.marine.copernicus.eu/?option=com_csw&task=results) |
| --- | --- |
| sea water potential temperature | NORTHWESTSHELF_ANALYSIS_FORECAST_PHY_004_013-TDS |
| sea surface temperature | SST_EUR_SST_L3S_NRT_OBSERVATIONS_010_009_a-TDS |
| sea water salinity | NORTHWESTSHELF_ANALYSIS_FORECAST_PHY_004_013-TDS |
| E and N water velocity | NORTHWESTSHELF_ANALYSIS_FORECAST_PHY_004_013-TDS |
| Wind speed | WIND_GLO_WIND_L4_REP_OBSERVATIONS_012_003-TDS |
| Chlorophyll | OCEANCOLOUR_ATL_CHL_L4_REP_OBSERVATIONS_009_098-TDS |
| Phytoplankton | NORTHWESTSHELF_REANALYSIS_BIO_004_011-TDS |

Appendix F: Rescaled distribution maps of the selected fish species, using the catch per unit of effort (CPUE), absence data for the occurrence of fish in the studied area, as well as environmental conditions (sea water potential temperature, sea surface temperature, sea water salinity, E and N water velocity, wind speed, chlorophyll, phytoplankton).


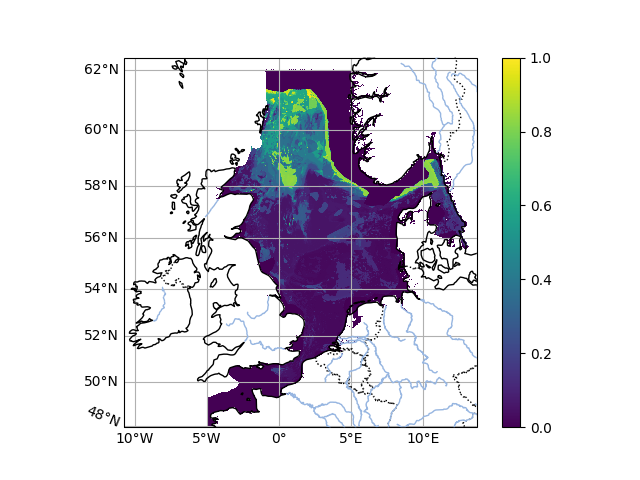

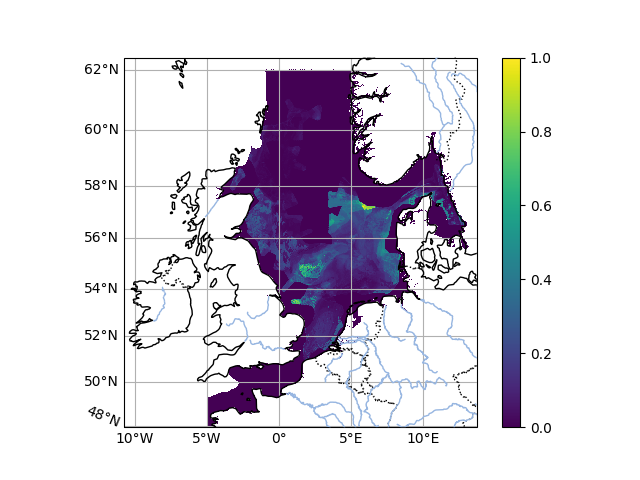


a. Fish – Saithe b. Fish – Sandele


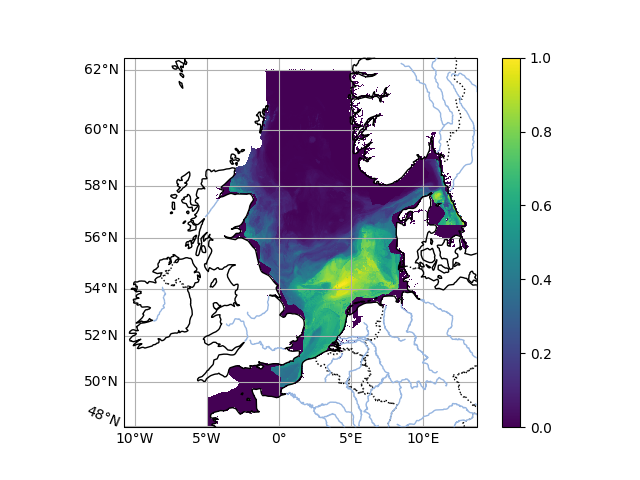

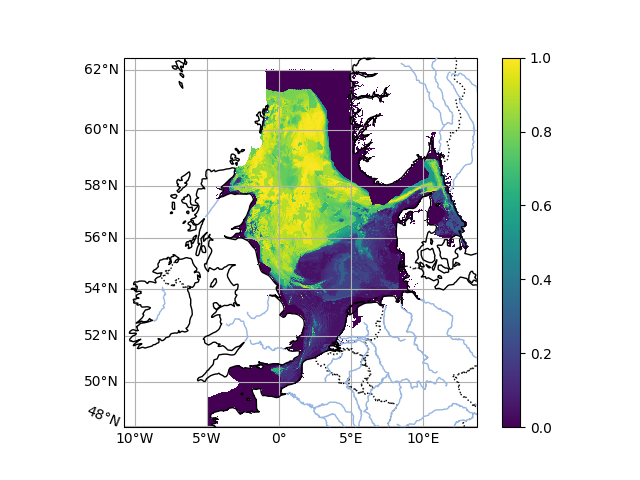


c. Fish – Sprat d. Fish – Haddock


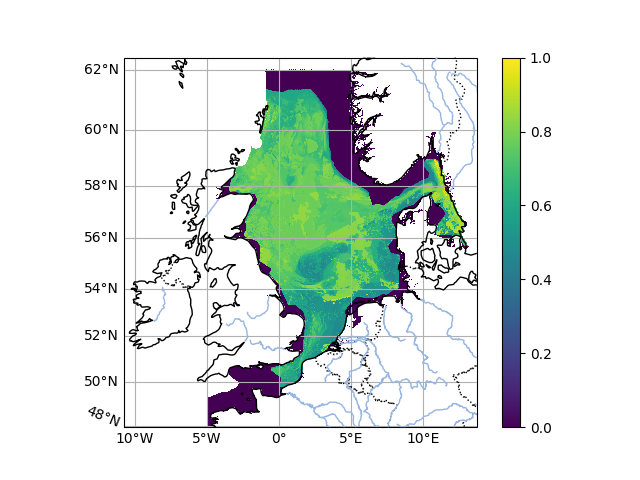

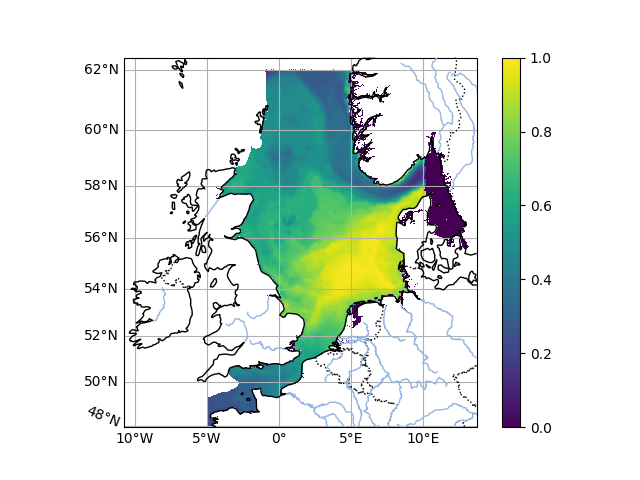


e. Fish – Whiting f. Marine Mammal – Harbor porpoise


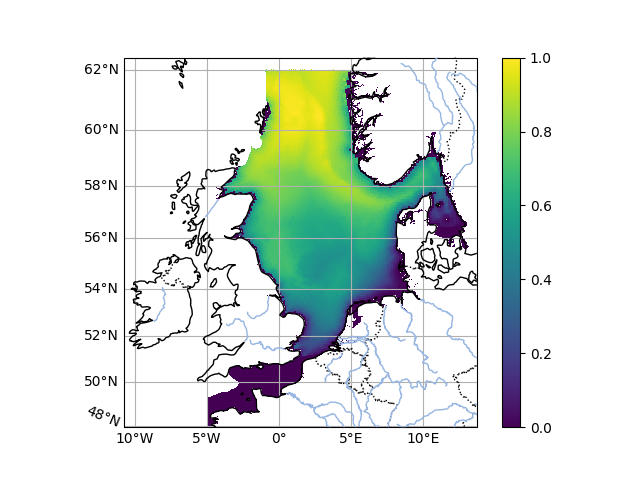

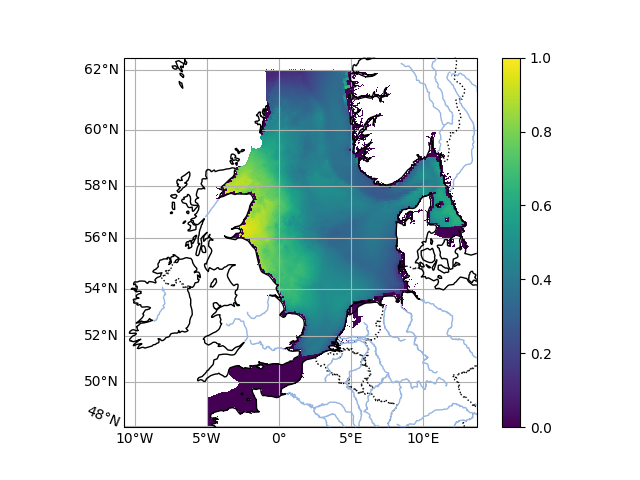


g. Marine birds – Fulmar h. Marine birds – Guillemot


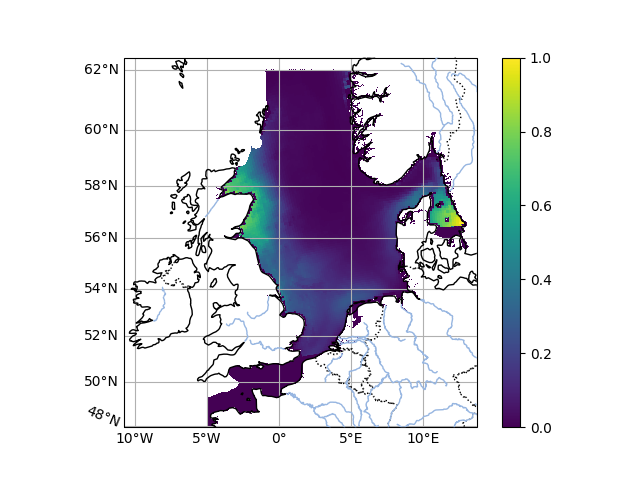

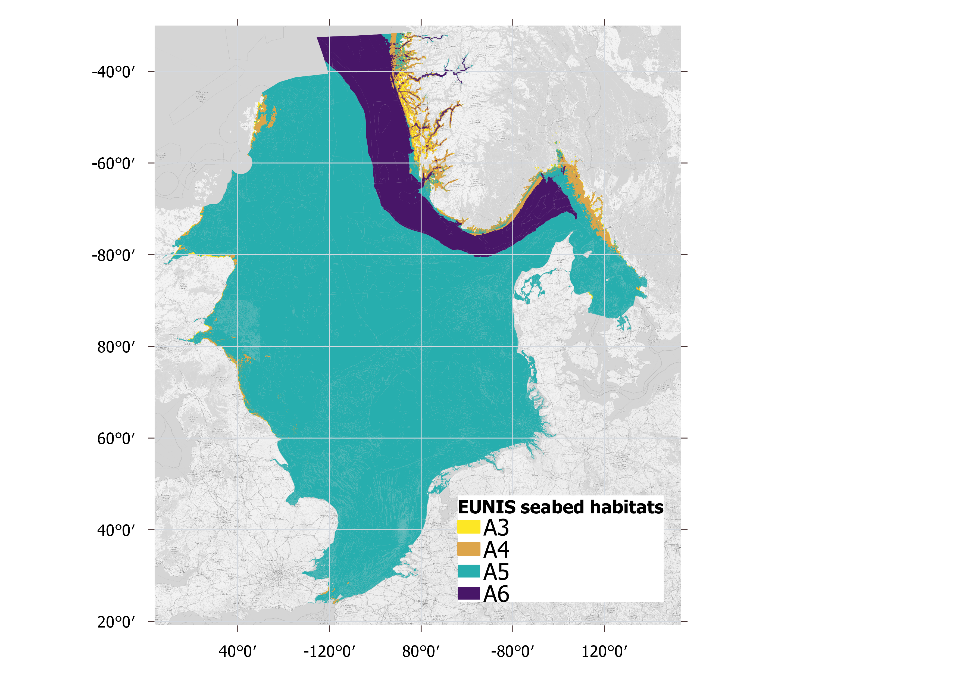


i. Marine birds – Razorbill j. Seabed habitats – A3, A4, A5, A6

Appendix G: The final environmental components sensitivity scores and the pressure weight scores resulted from the literature review (scientific articles and EIAs realized for OWFs), further validated through expert questionnaires.

We use the following definitions:

**Pressure**: defined as the mechanism through which an activity has an effect on any part of the ecosystem (<https://www.marlin.ac.uk/sensitivity/sensitivity_rationale>). Sources: 1) Marine Strategy Framework Directive; 2) SEANSE report “Comparison of North Sea SEA’s and EIA’s of maritime spatial plans and wind energy development”.

**Importance weight**: Not all pressures are equal in the three development phases. Therefore, we take into account the different importance weights of selected pressures. How do the different stages contribute to exert a given pressure? The range is presented in the table below:

Table 9. Scales used for importance of pressure weights

| 0 | Not relevant- the pressure is not relevant for this stage of offshore wind farm development. |
| --- | --- |
| 1 | Very low- should not influence or have very small impacts |
| 2 | Low |
| 3 | Medium - moderate influence on the species. |
| 4 | High |
| 5 | Very high - significantly influences the size or structure of the receptors. |
| Do not know | If the desired option is not available, we also welcome separate values with explanations. |

**Environmental components and their Sensitivities (s) to Pressures**: Sensitivity is defined as the likelihood of change when a pressure is applied to a feature (receptor) and is a function of the ability of the feature to tolerate or resist change and its ability to recover from the impact (<https://www.marlin.ac.uk/sensitivity/sensitivity_rationale>). The range of options is presented in the table below:

Table 10. Scales used for Sensitivity scores (negative impacts), with criteria based on: 1) adaptability, tolerance, recoverability of species to pressures (MarLIN): https://www.marlin.ac.uk/sensitivity/sensitivity_rationale

| 0 | Not sensitive - not impacted by the pressure |
| --- | --- |
| 1 | Very low - a full recoverability (5 years - few months), the effect does not affect the survival and viability of the individual or population. |
| 2 | Low |
| 3 | Medium - partial recoverability in 5 years, full recoverability in 10 years; not large scale fatal effects on the population but the viability of the species may be reduced; |
| 4 | High |
| 5 | Very high - only partial recovery is likely to occur after 10 years and full recovery might take up to 25 years; some individuals of the population may be killed by the impact and the viability of the population may be reduced. |
| Do not know | If the desired option is not available, we also welcome separate values with explanations. |

Table 11. Environmental components sensitivity and pressure weight mean scores – construction phase

|  | Construction phase | | | | | | | | | | | |
| --- | --- | --- | --- | --- | --- | --- | --- | --- | --- | --- | --- | --- |
| Pressures | ***Marine litter*** | ***Risk of contact with fuel or chemicals*** | ***Underwater noise*** | ***Collision (with vessels )*** | ***Barrier effect*** | ***Habitat loss*** | ***Food availability*** | ***Vibration*** | ***Sedimentation*** | ***Seabed disturbance*** | ***Sediment agitation*** | ***Release of sediment bound contaminants*** |
| *Importance weight* | 0.6 | 0.4 | 1 | 0.2 | 0.6 | 0.8 | 0.4 | 0.6 | 0.4 | 0.6 | 0.8 | 0.4 |
| *Environmental components and their sensitivities to pressures* |  | | | | | | | | | | | |
| *Cetaceans: harbour porpoise -(phocoena phocoena)* | 0.75 | 0.75 | 1 | 0.75 | 0.5 | 0.75 | 0.75 | 0.75 | 0 | 0 | 0.5 | 0.75 |
| *A 3: infralittoral rock and other hard substrata* | 0.5 | 0.75 | 0.75 | 0 | 0 | 0.5 | 0 | 0.5 | 0.75 | 0.5 | 0.5 | 0.5 |
| *A 4: circalittoral rock and other hard substrata* | 0.5 | 0.5 | 0.5 | 0 | 0 | 0.5 | 0 | 0.5 | 0.75 | 0.75 | 1 | 0.25 |
| *A 5: sublittoral sediment* | 0.5 | 0.75 | 0.75 | 0 | 0 | 0.75 | 0 | 0.5 | 0.5 | 0.5 | 0.75 | 0.25 |
| *Razorbill (alca torda)* | 0.5 | 0.75 | 0.5 | 0.5 | 1 | 0.75 | 0.75 | 0 | 0.25 | 0 | 0 | 0.5 |
| *Fulmar (fulmarus glacialis)* | 0.75 | 0.5 | 0.5 | 0.25 | 1 | 1 | 0.75 | 0 | 0 | 0 | 0 | 0.5 |
| *Guillemot (uria aalge)* | 0.5 | 0.75 | 0.5 | 0.5 | 1 | 0.75 | 0.75 | 0 | 0.25 | 0 | 0 | 0.5 |
| *Haddock (melanogrammus aeglefinus)* | 0.75 | 0.5 | 1 | 0 | 0.25 | 0.5 | 0.25 | 0.75 | 0.5 | 0.5 | 0.5 | 0.5 |
| *Sandeel (ammodytes tobianus)* | 0.25 | 0.25 | 1 | 0 | 0.25 | 1 | 0.25 | 0.75 | 1 | 1 | 0.5 | 0.5 |
| *Whiting (merlangius merlangus)* | 0.5 | 0.5 | 0.75 | 0 | 0.25 | 0.5 | 0.25 | 0.75 | 0.5 | 0.5 | 0.5 | 0.5 |
| *Saithe pollock (pollachius virens)* | 0.25 | 0.25 | 0.75 | 0 | 0.25 | 0.5 | 0.25 | 0.75 | 0.75 | 0.5 | 0.5 | 0.5 |
| *Sprat (sprattus sprattus)* | 0.5 | 0.5 | 0.5 | 0 | 0.25 | 0.5 | 0.25 | 0.75 | 0.5 | 0.5 | 0.5 | 0.5 |

Table 12. Environmental components sensitivity and pressure weight mean scores – operation phase

|  | Operation phase | | | | | | | | | | | | | | |
| --- | --- | --- | --- | --- | --- | --- | --- | --- | --- | --- | --- | --- | --- | --- | --- |
| Pressures | ***Risk of contact with fuel or chemicals*** | ***Change in physiochemical water quality*** | ***Significant changes in thermal regime*** | ***Significant changes in electromagnetic field*** | ***Underwater noise*** | ***Collision (with turbines)*** | ***Barrier effect*** | ***Habitat loss*** | ***Vibration*** | ***Sedimentation*** | ***Seabed disturbance*** | ***Sediment agitation*** | ***Release of sediment bound contaminants*** | ***Change in the hydrodynamic regime*** | ***Heat effect due to cabling*** |
| *Importance weight* | 0.2 | 0.2 | 0.4 | 0.2 | 0.4 | 0.6 | 1 | 0.6 | 0.4 | 0.2 | 0.2 | 0.2 | 0.2 | 0.4 | 0.4 |
| *Environmental components and their sensitivities to pressures* |  | | | | | | | | | | | | | | |
| *Cetaceans: harbour porpoise -(phocoena phocoena)* | 0.75 | 0.25 | 0.75 | 0.5 | 1 | 0.25 | 0.5 | 0.75 | 0.75 | 0 | 0 | 0.5 | 0.75 | 0 | 0 |
| *A 3: infralittoral rock and other hard substrata* | 0.75 | 0.5 | 0.5 | 0.5 | 0.75 | 0 | 0 | 0.5 | 0.5 | 0.75 | 0.5 | 0.5 | 0.5 | 0.75 | 0.5 |
| *A 4: circalittoral rock and other hard substrata* | 0.5 | 0.25 | 0.5 | 0.5 | 0.5 | 0 | 0 | 0.5 | 0.5 | 0.75 | 0.75 | 1 | 0.25 | 0.5 | 0.5 |
| *A 5: sublittoral sediment* | 0.75 | 0.5 | 0.5 | 0.5 | 0.75 | 0 | 0 | 0.75 | 0.5 | 0.5 | 0.5 | 0.75 | 0.25 | 0.5 | 0.5 |
| *Razorbill (alca torda)* | 0.75 | 0 | 0.25 | 0.25 | 0.5 | 0.25 | 1 | 0.75 | 0 | 0.25 | 0 | 0 | 0.5 | 0 | 0 |
| *Fulmar (fulmarus glacialis)* | 0.5 | 0 | 0.25 | 0.25 | 0.5 | 0.25 | 1 | 1 | 0 | 0 | 0 | 0 | 0.5 | 0 | 0 |
| *Guillemot (uria aalge)* | 0.75 | 0 | 0.25 | 0.25 | 0.5 | 0.25 | 1 | 0.75 | 0 | 0.25 | 0 | 0 | 0.5 | 0 | 0 |
| *Haddock (melanogrammus aeglefinus)* | 0.5 | 0 | 0.25 | 0.5 | 1 | 0 | 0.25 | 0.5 | 0.75 | 0.5 | 0.5 | 0.5 | 0.5 | 0.25 | 0.5 |
| *Sandeel (ammodytes tobianus)* | 0.25 | 0 | 0.25 | 0.5 | 1 | 0 | 0.25 | 1 | 0.75 | 1 | 1 | 0.5 | 0.5 | 0.25 | 0.5 |
| *Whiting (merlangius merlangus)* | 0.5 | 0 | 0.25 | 0.25 | 0.75 | 0 | 0.25 | 0.5 | 0.75 | 0.5 | 0.5 | 0.5 | 0.5 | 0.25 | 0.5 |
| *Saithe pollock (pollachius virens)* | 0.25 | 0 | 0.25 | 0.25 | 0.75 | 0 | 0.25 | 0.5 | 0.75 | 0.75 | 0.5 | 0.5 | 0.5 | 0.25 | 0.5 |
| *Sprat (sprattus sprattus)* | 0.5 | 0 | 0.25 | 0.25 | 0.5 | 0 | 0.25 | 0.5 | 0.75 | 0.5 | 0.5 | 0.5 | 0.5 | 0.25 | 0.5 |

Table 13. Environmental components sensitivity and pressure weight mean scores – decommissioning phase

|  | Decommissioning phase | | | | | | | | | | | | |
| --- | --- | --- | --- | --- | --- | --- | --- | --- | --- | --- | --- | --- | --- |
| Pressures | ***Marine litter*** | ***Risk of contact with fuel or chemicals*** | | ***Underwater noise*** | | ***Barrier effect*** | ***Habitat loss*** | ***Food availability*** | ***Vibration*** | ***Sedimentation*** | ***Seabed disturbance*** | ***Sediment agitation*** | ***Release of sediment bound contaminants*** |
| *Importance weight* | 0.6 | 0.4 | | 0.8 | | 0.4 | 0.6 | 0.4 | 0.6 | 0.4 | 0.6 | 0.8 | 0.4 |
| *Environmental components and their sensitivities to pressures* |  | | | | | | | | | | | | |
| *Cetaceans: harbour porpoise -(phocoena phocoena)* | 0.75 | | 0.75 | | 1 | 0.5 | 0.75 | 0.75 | 0.75 | 0 | 0 | 0.5 | 0.75 |
| *A 3: infralittoral rock and other hard substrata* | 0.5 | 0.75 | | 0.75 | | 0 | 0.5 | 0 | 0.5 | 0.75 | 0.5 | 0.5 | 0.5 |
| *A 4: circalittoral rock and other hard substrata* | 0.5 | 0.5 | | 0.5 | | 0 | 0.5 | 0 | 0.5 | 0.75 | 0.75 | 1 | 0.25 |
| *A 5: sublittoral sediment* | 0.5 | 0.75 | | 0.75 | | 0 | 0.75 | 0 | 0.5 | 0.5 | 0.5 | 0.75 | 0.25 |
| *Razorbill (alca torda)* | 0.5 | 0.75 | | 0.5 | | 1 | 0.75 | 0.75 | 0 | 0.25 | 0 | 0 | 0.5 |
| *Fulmar (fulmarus glacialis)* | 0.75 | 0.5 | | 0.5 | | 1 | 1 | 0.75 | 0 | 0 | 0 | 0 | 0.5 |
| *Guillemot (uria aalge)* | 0.5 | 0.75 | | 0.5 | | 1 | 0.75 | 0.75 | 0 | 0.25 | 0 | 0 | 0.5 |
| *Haddock (melanogrammus aeglefinus)* | 0.75 | 0.5 | | 1 | | 0.25 | 0.5 | 0.25 | 0.75 | 0.5 | 0.5 | 0.5 | 0.5 |
| *Sandeel (ammodytes tobianus)* | 0.25 | 0.25 | | 1 | | 0.25 | 1 | 0.25 | 0.75 | 1 | 1 | 0.5 | 0.5 |
| *Whiting (merlangius merlangus)* | 0.5 | 0.5 | | 0.75 | | 0.25 | 0.5 | 0.25 | 0.75 | 0.5 | 0.5 | 0.5 | 0.5 |
| *Saithe pollock (pollachius virens)* | 0.25 | 0.25 | | 0.75 | | 0.25 | 0.5 | 0.25 | 0.75 | 0.75 | 0.5 | 0.5 | 0.5 |
| *Sprat (sprattus sprattus)* | 0.5 | 0.5 | | 0.5 | | 0.25 | 0.5 | 0.25 | 0.75 | 0.5 | 0.5 | 0.5 | 0.5 |
